# Effects of T2-high asthma heterogeneity and inhaled corticosteroid on airway and metabolic profiles: A multi-omic approach

**DOI:** 10.1515/jtim-2026-0001

**Published:** 2026-02-13

**Authors:** Yuting Duan, Zhixia Gu, Tingting Liu, Chuan Song, Ying Wang, Wenjun Wang, Ronghua Jin, Xi Wang, Yuanyuan Zhang, Kewu Huang

**Affiliations:** Department of Respiratory and Critical Care Medicine, Beijing Institute of Respiratory Medicine, Beijing Chao-Yang Hospital, Capital Medical University, Beijing, China; National Key Laboratory of Intelligent Tracking and Forecasting for Infectious Diseases, Beijing Ditan Hospital, Capital Medical University, Beijing, China; National Center for Infectious Diseases, Beijing Ditan Hospital, Capital Medical University, Beijing, China; Beijing Key Laboratory of Viral Infectious Diseases, Beijing Ditan Hospital, Capital Medical University, Beijing, China; Beijing Institute of Infectious Diseases, Beijing Ditan Hospital, Capital Medical University, Beijing, China; Changping Laboratory, Beijing, China; Department of Oncology, Capital Medical University, Beijing, China

**Keywords:** asthma, type 2 helper T Cells, inflammation, corticosteroids, lipid metabolism disorders, cytokines, microbiota

## Abstract

**Background and Objectives:**

Asthma represents a heterogeneous chronic respiratory condition. Type 2 (T2) inflammation is the most crucial pathological event in asthma. In terms of whether T2 inflammation is dominant or not, asthma can be classified into T2-high and T2-low asthma. Currently, there exists a significant gap in our understanding of the heterogeneity of treatment-naive T2-high asthma patients. Moreover, no studies have examined the impacts of inhaled corticosteroids (ICS) on the airway microenvironment and metabolism of T2-high asthma during the early stage of treatment. This study, by employing multi-omic techniques, investigated the pathophysiological features and heterogeneity of untreated T2-high asthma, as well as the effects of ICS treatment. This study provided more in-depth insights into the pathophysiological mechanisms underlying T2-high asthma heterogeneity.

**Methods:**

Thirty-one treatment-naive T2-high asthma patients and fourteen healthy individuals were enrolled in this study. On the basis of hierarchical clustering analysis of T2 inflammation markers, fractional exhaled nitric oxide (FeNO) level and blood eosinophil count (BEC), the T2-high asthma patients were divided into three subgroups in terms of FeNO levels (≤ 25 ppb, 26-50 ppb, and > 50 ppb). All asthma patients underwent asthma control scoring, pulmonary function tests, and FeNO measurement at baseline and during a regular 3-month follow-up. Induced sputum and plasma were collected. Other tests included 16S rRNA microbiome profiling of the induced sputum, Luminex xMAP immunoassays of cytokines, and plasma metabolomic analysis using Q-Exactive liquid chromatography-mass spectrometry (LC-MS/MS). Meanwhile, data from the healthy population were also harvested.

**Results:**

T2-high asthma patients differed significantly from healthy controls in terms of airway inflammatory cytokines, airway microbial community structure, and plasma metabolic profiles. At baseline, T2-high asthma patients with different FeNO levels exhibited remarkable similarities in clinical symptoms, pulmonary function indices, airway cytokines, airway microbial diversity, and metabolites. After treatment with ICS, symptoms improved in T2-high asthma patients. The levels of FeNO, blood eosinophils, and total immunoglobulin E (tIgE) decreased significantly, while pulmonary function did not show substantial improvement. Some indices of airway cytokines underwent changes. No differences were found in airway microbial diversity; however, the abundance of *Actinomyces* increased. Moreover, the levels of glycerophospholipids and arachidonic acid metabolites decreased. Differentially expressed metabolites were enriched in arachidonic acid metabolism. The effect of ICS treatment varied among different T2-high asthma subgroups.

**Conclusions:**

The airway local microenvironment and systemic metabolic profiles of treatment-naive T2-high asthma patients were distinctly different from those of healthy individuals. Limited heterogeneity was observed among patients stratified in terms of T2-inflammatory burden. ICS altered the airway microenvironment and rectified the lipid/arachidonic acid metabolic dysregulation. However, ICS effects varied across various T2-high subgroups.

## Introduction

Asthma represents a heterogeneous chronic respiratory disorder and is characterized by recurrent wheezing, coughing, dyspnea, and chest tightness. The disease is one of the most prevalent chronic respiratory conditions across the globe.^[1,2]^ In China, epidemiological data indicate that 4.2% of adults aged ≥ 20 years (approximately 45.7 million individuals) are afflicted by asthma, posing substantial diagnostic and therapeutic challenges: 71.2% went undiagnosed and merely 5.6% received standardized treatment.^[3]^ Despite significant therapeutic advances, the global burden of asthma has remained persistently high in recent years,^[4]^ underscoring the need for improved understanding of disease mechanisms and optimized treatment strategies.

Contemporary classification systems categorize asthma endotypes by type 2 (T2) inflammation predominance, distinguishing T2-high asthma from T2-low asthma.^[5,6]^ Mounting evidence demonstrates distinct inter-phenotype variations in airway microbiome composition, immune responses, and plasma metabolic profiles, revealing a multifaceted heterogeneity of asthma pathogenesis. These findings highlighted the need for more granular patient stratification, particularly the better understanding of the role of the respiratory tract microenvironment in disease mechanisms and treatment outcomes.

T2-high asthma, affecting 50%-70% of asthma patients,^[7,8]^ is characterized by type 2 helper T cell dysregulation and chronic airway inflammation. This endotype presents substantial clinical heterogeneity, encompassing a diverse spectrum of subtypes, including allergic, eosinophilic, and occupational asthma, and responds differently to treatment. Although inhaled corticosteroids (ICS) remain the cornerstone therapy for asthma, treatment response varies substantially across T2-high subtypes. While mild-to-moderate cases generally exhibit good response to ICS, with severe forms, particularly adult-onset eosinophilic asthma, the therapeutic benefit tends to be limited.^[9]^ This variability primarily stems from the complex, heterogeneous nature of the airway inflammatory microenvironment, which significantly influences both disease pathogenesis and therapeutic efficacy.

Despite these advances, significant knowledge gaps linger in T2-high asthma research. Previous multi-omic studies have primarily looked at T2-low and severe T2-high asthma phenotypes, but largely overlooking mild-to-moderate T2-high asthma, especially treatment-naive populations. The microbial-proteomic-metabolomic characteristics during initial T2-high asthma treatment remain poorly characterized. Furthermore, current omic studies are of cross-sectional nature and involve phenotypically heterogeneous cohorts with diverse ICS exposure histories, limiting insights into the longitudinal effects of ICS therapy. Although longitudinal cohort studies have included treatment-naive asthma patients, their failure to stratify by endotypes or adjust for confounding variables has restricted their insights into the early effects of ICS therapy on the T2-high asthma airway microenvironment.

To overcome these limitations, we designed a comprehensive multi-omic study to characterize the heterogeneity in treatment-naive T2-high asthma at diagnosis and assess longitudinal ICS treatment effects. We systematically analyzed induced sputum to identify bacterial communities and inflammatory mediators, which allowed for precise profiling of the airway microenvironment in T2-high asthma. Furthermore, we used plasma metabolic profiling to look into the relationship between plasma metabolites and airway immune responses. This study had dual objectives: (1) characterizing airway microbiome composition, inflammatory signatures, and plasma metabolic profiles in treatment-naive T2-high asthma, and (2) assessing ICS-induced pathophysiological modifications in this population. By utilizing this integrated multi-omic approach, we sought to elucidate the T2-high asthma heterogeneity in molecular term and inform the development of precision therapeutic strategies.

## Materials and methods

### Cohort design

This study was a single-center, prospective longitudinal cohort study. Asthma patients were prospectively enrolled from the Respiratory Outpatient Clinic at Beijing Chaoyang Hospital, Capital Medical University, from April 2023 to December 2024. Healthy controls were recruited from the Institutional Health Examination Center. The study (ClinicalTrials.gov NCT05937334) received ethics approval from Beijing Chaoyang Hospital (2023-KE-260-2), with all participants having provided written informed consent.

### Cohort population

This study included Chinese asthma patients aged 18 to 75 who were newly diagnosed with asthma, had not received ICS treatment, and satisfied the diagnostic criteria of T2-high asthma. Healthy controls were individuals aged 18 and older. According to the global initiative for asthma (GINA) guidelines, asthma patients were diagnosed based on typical clinical symptoms and signs of asthma, and candidates with other diseases presenting similar symptoms were excluded. ^[10]^ Reversible airflow limitation was confirmed by relevant findings, such as a positive result for bronchodilator test (forced expiratory volume in 1 second [FEV_1_] increased by > 12% and an absolute increase of > 200 mL after inhalation of a bronchodilator) or a positive result with bronchial provocation test (FEV_1_ decreased by ≥ 20% after inhalation of a provocation agent). According to the GINA guidelines and previous studies,^[10, 11, 12]^ asthma patients were considered to have T2-high asthma if they met one or more of the following baseline clinical criteria: blood eosinophil count (BEC) ≥ 150/μL, and/ or fractional exhaled nitric oxide (FeNO) ≥ 20 ppb, and/or sputum eosinophils ≥ 2%, and/ or allergen-driven. Exclusion criteria included: (1) presence of other lung diseases or severe, uncontrolled systemic diseases; (2) a history of surgery within the past three months; (3) pregnancy or lactation; (4) current smokers (those who had not quit smoking in the past year) or those with a smoking history of more than 10 pack-years and significant biomass exposure; (5) symptoms and signs of respiratory infection within the past 4 weeks, or treatment with systemic and intranasal antibiotics; (6) diseases requiring systemic or oral steroid therapy.

### Data collection

At baseline, we collected demographic (gender, age, and body mass index [BMI]), socioeconomic (education level), and clinical data (smoking history, and allergy history) from all participants (asthma patients and healthy controls). For asthma patients, we took additional information about family history, initial symptoms, and trigger types (allergic or non-allergic). All subjects received budesonide/ formoterol (160/4.5 μg) bid for 12 weeks.

Pulmonary function tests were conducted at baseline and 12-week follow-up for all participants, following American Thoracic Society/ European Respiratory Society guidelines.^[13]^ We recorded forced vital capacity (FVC), FVC percentage of the predicted value (FVC%pred), FEV_1_, FEV_1_ percentage of the predicted value (FEV_1_%pred), and FEV_1_/FVC ratio (FEV_1_/FVC). We evaluated asthma symptom control at baseline and during follow-up using asthma-related questionnaires, including the Asthma Control Test (ACT), Asthma Control Questionnaire-5 (ACQ-5), and Asthma Quality of Life Questionnaire (AQLQ). We measured FeNO before lung function tests on a nitric oxide analyzer (NIOX; Aerocrine AB, Stockholm, Sweden) at a flow rate of 50 mL/s, as recommended by American Thoracic Society (ATS).^[14]^ Venous blood samples were collected for the analysis of BEC, blood eosinophil percentage (B-EOS%), total immunoglobulin E (tIgE), inhalant-specific IgE (sIgE), and plasma metabolomic analysis.

Induced sputum samples were taken by trained personnel for cell count and asthma was classified, according to the extensively-accepted four-class method, into the following types: neutrophilic asthma (NA, sputum NEUT% ≥ 61%, sputum EOS% < 2%); eosinophilic asthma (EA, sputum EOS% ≥ 2%, sputum NEUT% < 61%); mixed granulocytic asthma (MA, sputum NEUT% ≥ 61%, sputum EOS% ≥ 2%); and paucigranulocytic asthma (PA, sputum NEUT% < 61%, sputum EOS% < 2%).^[15,16]^

### Induced sputum collection and DNA extraction

Sputum samples from both healthy controls and asthma patients were collected at baseline and during follow-up. After quality control of the specimens, each sample was divided into two parts. One portion of the sputum sample was processed by trained professionals for induced sputum cell counting and classification. The other portion was used for DNA extraction by using the MagaBio Pathogen DNA/ RNA Purification Kit (BioFlux, Bioer Technology, China). DNA concentration, purity, integrity, and size were measured by using Nanodrop (Thermo Fisher Scientific, USA) and 1.0% agarose gel electrophoresis.

### Sequencing of bacterial 16S ribosomal RNA (rRNA) gene in induced sputum

The bacterial 16S rRNA gene sequences containing the variable regions V3-V4 were amplified by using primers 341F (5’-CCTACGGGNGGCWGCAG-3’) and 805R (5’-GACTACHVGGGTATCTAATCC-3’) and the Q5 High-Fidelity 2X Master Mix (New England BioLabs, USA). The products were purified using magnetic beads (AMPure XP, Beckman Coulter Inc., USA), and each sample was quantitatively analyzed by using a fluorescence spectrometer (Qubit 2.0, ThermoFisher Scientific, USA). The samples were mixed at the same starting mass, and then further purified with magnetic beads. The final sequencing pool concentration was determined by qPCR using a library quantification kit (KAPA Biosystems, USA). Sequencing was carried out on an Illumina Novaseq sequencer (Illumina, USA) using the Illumina PE 250 platform by Beijing Novogene Technology Co.

High-throughput 16S rRNA sequencing raw fastq files were de-multiplexed and quality filtered using QIIME (version 2022.8.0). Dada2 was used to trim adapter sequences, merge paired ends, eliminate chimeras, and generate amplicon sequence variants (ASVs) to reduce noise. Each 16S rRNA gene sequence was taxonomically analyzed using QIIME (version 2022.8.0) and the result was compared with the SILVA rRNA database, with a confidence threshold set at 70%.

### Multiplex immunoassay for cytokines and chemokines

By using the Luminex xMAP technology, 45 cytokines and chemokines in the induced sputum supernatants from asthma patients and healthy controls were measured on the Bio-Plex platform (Bio-Rad, USA) with the pre-configured 45-plex ProcartaPlex Immunoassay Kit (Invitrogen, Thermo Fisher Scientific, USA). Since the concentrations of more than 20% of samples were below the detection limit for certain cytokines, 14 cytokines were excluded from the analysis. These cytokines included granulocyte-macrophage colony stimulating factor (GMCSF), growth-related oncogene-α (GRO-α) (CXCL1), interferon-γ (IFN-γ), interleukin-2 (IL-2), IL-12p70, IL-13, IL-17A, IL-27, IL-5, IL-9, monocyte chemotactic protein-1 (MCP-1) (CCL2), macrophage-derived chemokine (MDC) (CCL22), stromal-derived factor-1α (SDF-1α) (CXCL12), and tumor necrosis factor-α (TNF-α). Additionally, 5 cytokines (IL-4, IL-7, monokine induced by interferon-gamma (MIG) [CXCL9], TNF-β, and thymic stromal lymphopoietin (TSLP)) in the healthy control group were excluded. Therefore, the baseline data of 26 cytokines were retained for analysis, as their concentrations were within the standard curve range and their sample detection rates were above 80%. These cytokines included CCL17, Eotaxin (CCL11), Eotaxin-2 (CCL24), fibroblast growth factor-2 (FGF-2), G-CSF, IFN-α, IL-10, IL-15, IL-18, IL-1RA, IL-1α, IL-1β, IL-21, IL-22, IL-23, IL-31, IL-33, IL-6, IL-8, IP-10 (CXCL10), macrophage inflammatory protein-1α (MIP-1α) (CCL3), MIP-1β (CCL4), matrix metalloproteinase-9 (MMP-9), RANTES (CCL5), TNF-RI, and TNF-RII. In the asthma group, 31 cytokines were retained for analysis, including CCL17, Eotaxin (CCL11), Eotaxin-2 (CCL24), FGF-2, G-CSF, IFN-α, IL-10, IL-15, IL-18, IL-1RA, IL-1α, IL-1β, IL-21, IL-22, IL-23, IL-31, IL-33, IL-4, IL-6, IL-7, IL-8, IP-10 (CXCL10), MIG (CXCL9), MIP-1α (CCL3), MIP-1β (CCL4), MMP-9, RANTES (CCL5), TNF-RI, TNF-RII, TNF-β, and TSLP. For longitudinal comparisons of cytokines at different time points, based on a sample detection rate higher than 80% for each time point, 25 cytokines were retained that had valid sample numbers at every time point. These cytokines included Eotaxin-2, FGF-2, G-CSF, IL-33, MIG, MMP-9, CCL17, TNF-RII, TSLP, IL-1β, IL-6, IL-18, IL-21, IL-22, IL-23, IFN-α, IL-31, IL-15, IL-1α, IL-1RA, IL-7, IL-8, IP-10, MIP-1α, and MIP-1β. All assays were performed by following the manufacturer‘s instructions.

### Plasma metabolomic analysis

Plasma metabolites of participants were analyzed using a Q Exactive liquid chromatography-mass spectrometry (LC-MS/MS) system (Thermo Scientific, USA). The raw data were converted into mzXML format (xcms input file format) using Proteowizard software (v3.0.8789), and peak identification, peak filtration, and peak alignment were performed using the XCMS package in R, yielding a data matrix with information on mass-to-charge ratio (m/z), retention time, and peak intensity. Both positive and negative ion modes were used to detect precursor molecules, and data were exported to Excel for subsequent analysis.

### Bioinformatic and statistical analyses

Statistical analysis and visualization were performed by using R software (version 4.2.3), RStudio (version 2024.09.0-375), and GraphPad Prism 10. A significance level of *P* < 0.05 (two-tailed test) was used for all statistical tests. Categorical data were expressed as percentages and analyzed using chi-square tests (Fisher‘s exact test for *n* < 40). Continuous data were presented as means ± standard deviation (SD) (for normally distributed data) or median and interquartile range (for non-normally distributed data). For normally distributed data, comparisons between two independent groups were made using the two-sample *t*-test, and comparisons between multiple groups were conducted using ANOVA, followed by Tukey’s HSD test for post-hoc comparisons. For skewed data, the Wilcoxon rank-sum test was employed for comparisons between two groups, and the Kruskal-Wallis test was utilized for comparisons among multiple groups, with Dunn‘s test used for post-hoc comparisons. Repeated measures within groups at different time points were analyzed using the Wilcoxon Signed-Rank Test. Hierarchical clustering analysis was performed using Ward‘s method to generate a dendrogram and determine the number of clusters. Spearman correlation analysis was performed to evaluate the association between the different differential indicators.

For microbiome data, the total abundance of each sample was calculated and visualized with a bar chart, with a reference line indicating the rarefaction depth. Samples were selected based on the predefined rarefaction depth, and rarefaction was performed using the GUniFrac package. Alpha diversity was calculated, including species richness (Richness, Chao1 index, abundance-based coverage estimator (ACE) index, and Sobs index) and species diversity (Shannon index and Simpson index). Beta diversity was assessed using principal component analysis (PCA) and principal coordinates analysis (PCoA). Differential microbiomes were then compared.

Metabolomic analysis was performed using the R package MetaboAnalystR. Upon quality control (QC) and batch correction, data normalization was performed, followed by statistical analysis of metabolite concentrations (bar charts and heatmaps). Unsupervised dimensionality reduction (*e.g*., PCA) and feature metabolite selection (*e.g*., partial least squares discriminant analysis (PLSDA), univariate analysis, volcano plots, and machine learning) were performed. Pathway analysis (*e.g*., enrichment analysis, topological analysis, and pathway diagrams) was conducted for the selected metabolites. All data analyses were performed on the Bioincloud platform (https://www.bioincloud.tech/).^[17]^

## Results

### Multi-omic profiling of T2-high asthma patients

All thirty-one treatment-naive T2-high asthma patients meeting inclusion criteria completed ICS treatment and follow-up. Meanwhile, 14 healthy individuals were enrolled as controls. Supplementary Figure S1 illustrates the study‘s inclusion and exclusion criteria for asthma patients. Table 1 summarizes the demographic characteristics of both the asthma (AS) and healthy control (HC) groups. There were no significant differences in gender, age, BMI, and educational level between the HC group and the AS group. The proportion of females was higher than that of males in both groups (57.1% *vs*. 64.5%). In both groups, a substantial proportion of individuals received a college education or higher (64.3% *vs*. 74.2%). Moreover, the majority of the enrolled participants had never smoked (85.7% *vs*. 83.9%). Over half of asthma patients (18/31, 58.1%) had allergic comorbidities, while 25.8% (8/31) reported familial asthma history. Cough, wheezing, and chest tightness were the most common clinical symptoms. Meanwhile, dust mites, pets, and pollen were common allergens. Sputum cell classification revealed a predominance of eosinophilic asthma (21/28, 75.0%), with paucigranulocytic/neutrophilic asthma patients accounting for 25.0% (7/28). There were no cases of mixed granulocytic asthma. In addition, the sputum cell count was insufficient in 3 patients, rendering the data unavailable for these individuals.

**Table 1:** Characteristics of asthma patients and healthy controls

Parameter	Asthmatic patients (*n* = 31)	Healthy controls (*n* = 14)	*P*
Gender			0.744
Male, *n* (%)	11 (35.5)	6 (42.9)	
Female, *n* (%)	20 (64.5)	8 (57.1)	
Age, year	44.32 ± 13.06	40.50 ± 11.16	0.322
BMI, kg/m^2^	25.42 ± 3.89	24.28 ± 4.39	0.371
Degree of education			0.617
Elementary school or below	1 (3.2)	1 (7.1)	
Junior and high school	7 (22.6)	4 (28.6)	
College	23 (74.2)	9 (64.3)	
Cigarette smoking			0.999
Never-smoker	26 (83.9)	12 (85.7)	
Passive smoking at home	17 (54.8)	0 (0)	
Ever-smoker	5 (19.4)	2 (14.3)	
Allergic disease			
All	18 (58.1)	N/A	
Allergic rhinitis	14 (45.2)	N/A	
Allergic conjunctivitis	3 (9.7)	N/A	
Urticaria	4 (12.9)	N/A	
Eczema	6 (19.4)	N/A	
Family History	8 (25.8)	N/A	
Symptoms			
Chest tightness	18 (58.1)	N/A	
Breathlessness	3 (9.7)	N/A	
Wheezing	8 (25.8)	N/A	
Cough	22 (71.0)	N/A	
Predisposing triggers			
Respiratory infections	10 (32.3)	N/A	
Dust mite	7 (22.6)	N/A	
Pet	5 (16.1)	N/A	
Pollen	4 (12.9)	N/A	
Sputum-based phenotypes			
Eosinophilic asthma	21 (75.0)	N/A	
Neutrophilic asthma	1 (3.6)	N/A	
Paucigranulocytic asthma	6 (21.4)	N/A	
Mixed granulocytic asthma	0 (0)	N/A	
B-EOS%, %	3.9 (1.6-5.7)	1.7 (0.8-2.6)	0.015
BEC, 10^9^/L	0.24 (0.09-0.33)	0.09 (0.04-0.13)	0.010
tIgE, KU/L	146 (17-121)	111 (28-155)	0.371
sIgE, KU/L	3.82 (0.05-4.38)	1.62 (0.07-0.27)	0.941
FeNO, ppb	47.0 (18.5-62.5)	18.1 (14.0-20.8)	0.003
FVC, L	3.94 ± 0.89	3.55 ± 0.77	0.147
FVC%pred, %	111.5 ± 11.2	99.7 ± 9.9	0.001
FEV_1_, L	3.00 ± 0.80	2.93 ± 0.68	0.754
FEV_1_%pred, %	100.7 ± 14.3	97.2 ± 10.0	0.265
FEV_1_/FVC, %	75.9 ± 7.9	82.4 ± 3.8	0.002

Values are mean ± SEM or Median (25th-75th percentile) or *n* (%) unless otherwise stated. B-EOS%: blood eosinophil percent; BEC: blood eosinophil count; tIgE: total immunoglobulin E; sIgE: specific immunoglobulin E; FeNO: fractional exhaled nitric oxide; FVC: forced vital capacity; FVC%pred: forced vital capacity in percent of the predicted value; FEV_1_: forced expiratory volume in 1 second; FEV_1_% pred: forced expiratory volume in one second in percent of the predicted value; FEV_1_/FVC: ratio of forced expiratory volume in 1 second to forced vital capacity.

**Figure 1:**
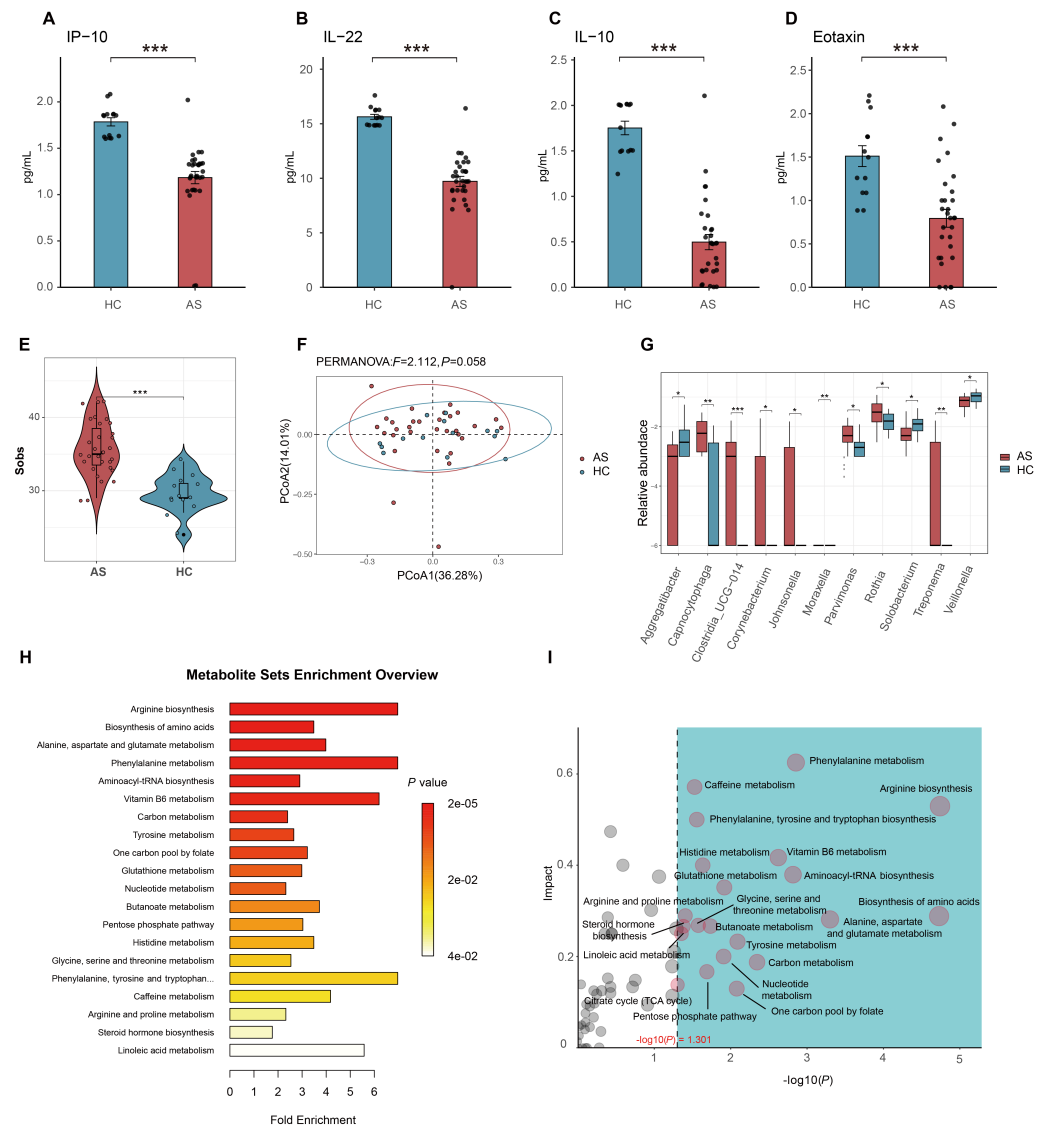
Differences in sputum cytokines, airway microbiota, and plasma metabolites between T2-high asthma patients and healthy controls at baseline. (A-D) Differential sputum cytokines between asthma patients and healthy controls (IP-10/IL-22/IL-10/Eotaxin). (E) Comparison of the Sobs index of airway microbiota α-diversity between asthma patients and healthy controls. (F) PCoA of airway microbiota β-diversity between asthma patients and healthy controls. (G) Taxonomic differences at genus level between asthma patients and healthy controls. (H) Metabolite enrichment analysis. The x-axis represents the enrichment ratio, calculated as the observed number of metabolites/theoretical number of metabolites in the metabolic pathway. The size of the *P*-value is denoted by different colors, with darker shades indicating smaller *P*-values. (I) Results of metabolite enrichment and topological analysis. The x-axis represents the P-value, with the blue area indicating statistical significance (*P* < 0.05); the y-axis represents the impact from the topological analysis. ^*^P < 0.05; ^**^P < 0.01; ^***^P < 0.001. HC: healthy control; AS: asthma patients; IP: interferon-γ-inducible protein; **IL: interleukin. PcoA: principal coordinates analysis.**

The AS group exhibited significantly elevated BEC, B-EOS%, and FeNO levels compared to HC group (Table 1). While FVC%pred was higher in AS patients, their FEV_1_/FVC was significantly reduced relative to controls. The concentrations of G-CSF, IL-15, IL1-RA, and TNF-RI were higher in the AS group than in the HC group. The levels of eotaxin, IFN-α, IL-10, IL-18, IL-22, IL-23, and IP-10 were lower in the AS group than in the HC group (Table 2, Figure 1A-1D).

**Table 2:** Sputum cytokines compared across asthma patients and healthy controls

Cytokines*	Asthmatic patients (*n* = 31)	Healthy controls (*n* = 14)	*P*
CCL17	0.21 (0.16-0.28)	0.20 (0.14-0.26)	0.659
Eotaxin	0.79 (0.34-1.05)	1.51 (1.13-1.73)	< 0.001
Eotaxin-2	1.09 (1.00-1.23)	0.94 (0.83-1.02)	0.067
FGF-2	20.22 (5.89-10.09)	11.06 (6.80-13.76)	0.202
G-CSF	24.29 (6.39-28.95)	23.62 (21.76-24.30)	0.022
IFN-α	0.39 (0.22-0.49)	0.63 (0.46-0.84)	0.020
IL-10	0.50 (0.18-0.64)	1.75 (1.50-2.00)	< 0.001
IL-15	3.90 (2.84-4.96)	2.13 (1.37-2.79)	0.001
IL-18	29.52 (6.83-38.88)	84.64 (24.25-74.67)	0.020
IL-1RA	49.96 (28.21-67.72)	36.13 (6.02-43.05)	0.011
IL-1α	22.68 (8.91-26.80)	38.02 (11.34-31.82)	0.816
IL-1β	172.00 (61.70-228.30)	182.80 (48.70-235.10)	0.816
IL-21	7.82 (4.57-10.35)	8.83 (5.77-11.01)	0.580
IL-22	9.72 (8.88-10.82)	15.64 (14.89-16.24)	< 0.001
IL-23	4.29 (1.37-7.49)	6.90 (5.36-8.15)	0.010
IL-31	19.83 (7.42-27.59)	19.41 (15.09-21.33)	0.370
IL-33	13.07 (11.98-14.35)	13.73 (12.31-14.48)	0.547
IL-6	10.43 (6.21-11.55)	10.75 (8.88-10.89)	0.102
IL-8	153.50 (46.80-196.60)	76.90 (33.90-80.20)	0.114
IP-10	1.18 (1.12-1.32)	1.78 (1.62-1.85)	< 0.001
MIP-1α	2.71 (0.45-3.03)	2.84 (0.78-2.64)	0.371
MIP-1β	23.92 (3.88-35.14)	17.63(3.73-15.23)	0.377
MMP-9	545.50 (246.90-743.10)	690.20 (364.90-809.40)	0.159
RANTES	0.57 (0.50-0.64)	0.62 (0.57-0.63)	0.437
TNF-RI	184.80 (91.30-261.20)	99.60 (88.00-122.60)	0.003
TNF-RII	0.53 (0.50-0.64)	0.52 (0.25-0.84)	0.919

Values are Median (25th-75th percentile) unless otherwise stated. *The expression levels of other cytokines are presented in pg/mL, while that of IL-RA is in ng/mL. CCL: chemokine (C-C motif) ligand; FGF: fibroblast growth factor; G-CSF: granulocyte colony-stimulating factor; IFN: interferon; IL: interleukin; IP: interferon-γ-inducible protein; MIP: macrophage inflammatory protein; MMP: matrix metalloproteinase; TNF-R: tumor necrosis factor receptor.

Except that the number of airway microbial species in the AS group was significantly higher than that in the HC group (Figure 1E, Supplementary Figure S2A-S2C), no significant differences were observed between the two groups in terms of microbial alpha diversity (Supplementary Figure S2D-S2E) and beta diversity (Figure 1F, Supplementary Figure S2F). There were differences in the abundances of 11 bacterial genera between the AS group and the HC group (Figure 1G), and most of these abundances showed a negative correlation with inflammatory factors (Supplementary Figure S3A-S3B). There also existed differences in the proportions of the top ten bacteria at the genus level (Supplementary Figure S2G). Plasma metabolomic analysis revealed that the AS group and the HC group had completely distinctly differential metabolic features (Supplementary Figure S2H-S2M).

### Multi-omic profiling in T2-high asthma subgroups

**Figure 2:**
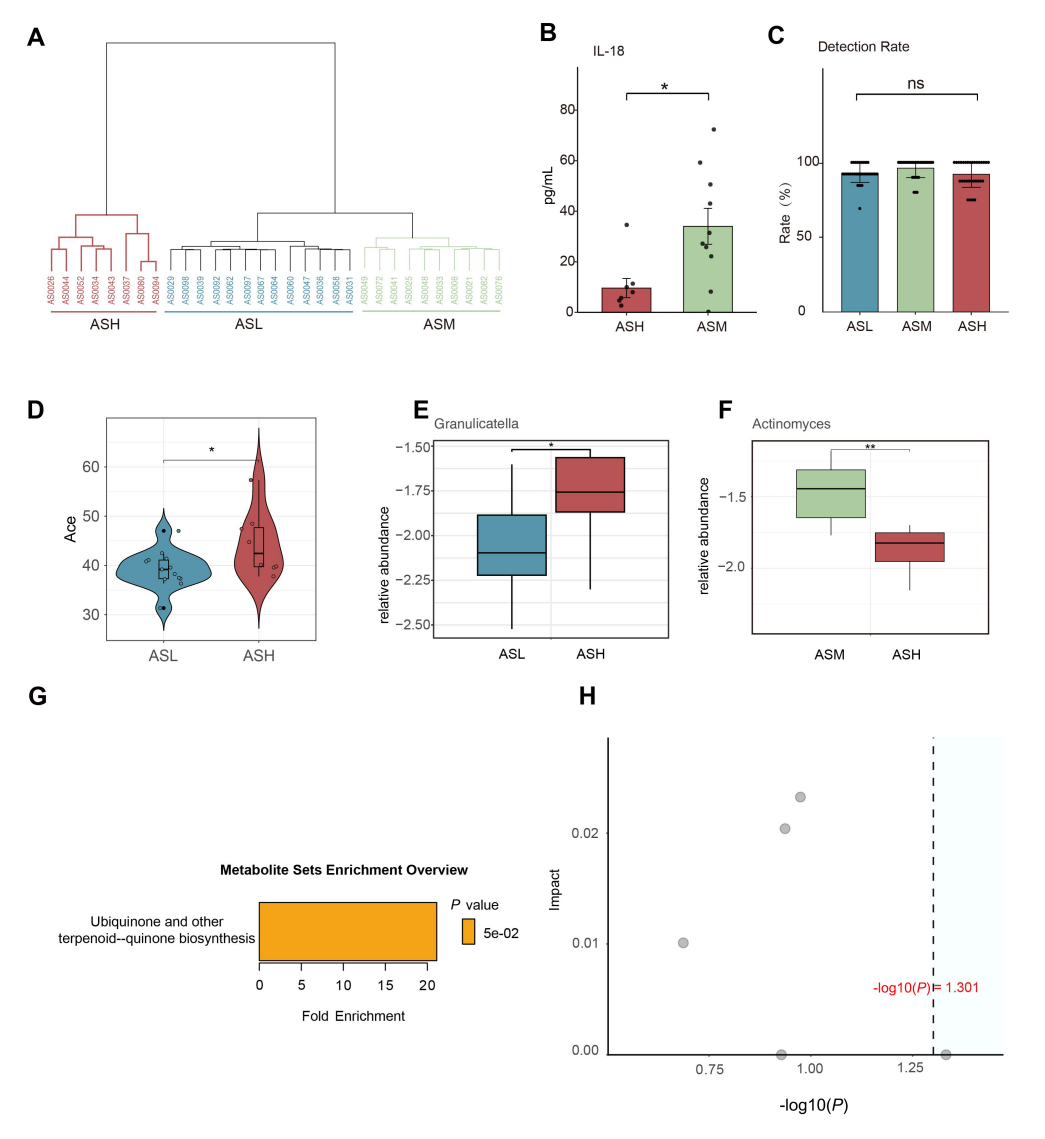
Differences in sputum cytokines, airway microbiota, and plasma metabolites at baseline across FeNO subgroups. (A) Hierarchical clustering of T2-high asthma patients included in the study. All participants were categorized into the ASL group (blue), ASM group (yellow), and ASH group (red). (B) Cytokine markers with significant differences between subgroups in pairwise comparisons. (C) Comparison of cytokine detection rates among the three subgroups. (D) Difference in ACE index between the ASL and ASH subgroups. (E-F) Differential microbiota between the two subgroups. (G) Metabolite enrichment analysis. (H) Results of metabolite enrichment and topological analysis. ns*P* > 0.05; ^*^P < 0.05; ^**^P < 0.01; ASL: FeNO level low; ASM: FeNO level moderate; ASH: FeNO level high.

We performed hierarchical clustering for 31 T2-high asthma patients using two clinical biomarkers (FeNO and BEC). All patients were stratified into three distinct subgroups in terms of baseline FeNO levels, *i.e*. low [asthma FeNO level low (ASL), *n* = 13; FeNO ≤ 25 ppb], moderate [asthma FeNO level moderate (ASM), *n* = 10; 26-50 ppb], and high [asthma FeNO level high (ASH), *n* = 8; FeNO > 50 ppb] FeNO groups (Figure 2A, Supplementary Figure S1).

At baseline, the three groups showed significant differences in ACQ scores, BEC, B-EOS%, and tIgE levels, but comparable pulmonary function profiles, ACT scores, and AQLQ scores (Table 3). The three groups exhibited similar levels of all 31 cytokines measured in sputum supernatants (Supplementary Table S1). Among the groups, only IL-18 levels differed significantly between the ASH and ASM groups (Figure 2B). No differences were found in cytokine detection rates across the three groups (Figure 2C).

**Table 3:** Baseline clinical characteristics of three stable FeNO subgroups

Parameter	ASH (*n* = 8)	ASM (*n* = 10)	ASL (*n* = 13)	Pall	PHM	PHL	PML
ACT, score	17.25 ± 2.60	19.10 ± 1.37	18.77 ± 2.74	0.289	0.114	0.222	0.710
ACQ, score	1.90 (0.90-2.80)	0.60 (0.10-1.00)	0.60 (0-1.00)	0.047	0.039	0.028	0.874
AQLQ, score	5.73 ± 0.59	5.73 ± 0.83	5.95 ± 0.90	0.495	0.998	0.512	0.369
Symptoms	4.94 ± 1.07	5.86 ± 0.66	5.81 ± 1.02	0.118	0.055	0.095	0.555
Activity limitation	6.14 ± 0.58	5.64 ± 1.22	5.99 ± 1.00	0.686	0.448	0.999	0.494
Emotional function	5.88 ± 0.99	5.90 ± 0.96	6.20 ± 0.93	0.610	0.999	0.437	0.414
Environmental stimuli	5.97 ± 1.05	5.53 ± 1.07	5.79 ± 1.19	0.651	0.392	0.712	0.574
B-EOS%, %	8.6 (5.9-10.1)	2.7 (1.6-3.7)	2.0 (1.1-2.9)	< 0.001	0.002	< 0.001	0.419
BEC, 10^9^/L	0.52 (0.33-0.56)	0.16 (0.09-0.17)	0.13 (0.07-0.18)	< 0.001	0.002	< 0.001	0.617
tIgE, KU/L	250 (100-250)	205 (55-111)	37 (13-54)	0.009	0.307	0.004	0.051
sIgE, KU/L	7.06 (0.08-5.36)	3.59 (0.04-7.99)	2.02 (0.05-1.27)	0.379	0.503	0.137	0.755
FeNO, ppb	111.0 (90.3-127.5)	35.1 (30.3-38.8)	16.8 (14.0-19.0)	< 0.001	< 0.001	< 0.001	< 0.001
FVC, L	3.55 ± 0.78	3.94 ± 0.89	4.17 ± 0.94	0.195	0.338	0.123	0.562
FVC%pred, %	111.3 ± 13.4	111.4 ± 10.6	111.7 ± 11.1	0.770	0.992	0.951	0.949
FEV_1_, L	2.48 ± 0.74	3.07 ± 0.66	3.27 ± 0.82	0.107	0.103	0.038	0.514
FEV_1_%pred, %	92.2 ± 18.7	103.4 ± 11.9	103.7 ± 11.6	0.285	0.307	0.103	0.952
FEV_1_/FVC, %	69.4 ± 10.9	78.1 ± 4.0	78.3 ± 6.0	0.111	0.069	0.062	0.923

Values are mean ± SEM or Median (25th-75th percentile) unless otherwise stated. ACT: Asthma Control Test; ACQ: Asthma Control Questionnaire; AQLQ: Asthma Quality of Life Questionnaire; B-EOS%: blood eosinophil percent; BEC: blood eosinophil count; tIgE: total immunoglobulin E; sIgE: specific immunoglobulin E; FeNO: fractional exhaled nitric oxide; FVC: forced vital capacity; FVC%pred: forced vital capacity in percent of the predicted value; FEV_1_: forced expiratory volume in 1 second; FEV_1_%pred: forced expiratory volume in one second in percent of the predicted value; FEV_1_/FVC: ratio of forced expiratory volume in 1 second to forced vital capacity.

No significant differences in airway microbiota alpha-diversity or beta-diversity were revealed among the three groups (Supplementary Figure S4A-S4H). No differentially abundant bacterial taxa were identified among the three groups. Comparison of the top ten most abundant bacterial genera among the three groups revealed that the ASH group had the lowest abundance of *Streptococcus* and *Actinomyces*, but the highest abundance of *Fusobacterium*, *Neisseria*, and *Haemophilus*. However, none of these differences reached statistical significance (Supplementary Figure S4I). Significant differences in ACE index values were observed between the ASH and ASL groups (Figure 2D). Differential abundance analysis revealed significantly higher levels of *Granulicatella* in the ASH group compared to ASL, while *Actinomyces* showed significantly lower abundance in ASH relative to ASM (Figure 2E-2F). There were no significant differences in metabolic profiles among the three groups (Supplementary Figure S5A-S5F). Kyoto Encyclopedia of Genes and Genomes (KEGG) pathway enrichment analysis revealed that differentially abundant metabolites were significantly enriched in ubiquinone and other terpenoid-quinone biosynthesis pathways (*P* = 0.047, Figure 2G). Nonetheless, topological analysis showed these metabolites exerted negligible impact on pathway flux (impact score = 0) (Figure 2H).

### Multi-omic dynamics in asthma patients post-ICS treatment

We first assessed changes in asthma control and lung function profiles following ICS treatment in all T2-high patients. Asthma control was defined as an ACT score ≥20 post-treatment. The majority of patients achieved asthma control after ICS treatment (29/31, 93.6%). The two uncontrolled cases included an ASH patient with persistently high FeNO (127 ppb) and unchanged ACT score (18), and an ASM patient with moderate FeNO (40 ppb) whose ACT score improved marginally from 17 to 19. Both patients suffered from eosinophilic asthma with allergen-driven pathophysiology. The 29 patients who attained asthma control were subjected to subsequent analysis (Supplementary Figure S1). Following treatment, significant improvements were observed in both ACT scores and AQLQ scores, while ACQ scores dropped significantly. FeNO, BEC, B-EOS%, and tIgE were all significantly lowered. However, no significant improvement was found in the lung function profile (Table 4).

**Table 4:** Clinical characteristics compared across baseline and follow-up of the enrolled initial-diagnosis asthma patients (*n* = 29)

Parameter	Baseline	Follow-up	Mean improvement from baseline	*P*
ACT, score	18.55 ± 2.46	23.14 ± 1.13	4.59 ± 2.50	< 0.001
ACQ, score	0.80 (0-1.20)	0.30 (0-0.40)	-0.40 (-1.10-0.10)	0.010
AQLQ, score	5.90 ± 0.74	6.23 ± 0.73	0.27 (0.06-0.67)	0.001
Symptoms	5.69 ± 0.95	6.32 ± 0.45	0.34 (-0.04-1.41)	0.002
Activity limitation	6.01 ± 0.93	6.26 ± 0.79	0.19 (-0.09-0.55)	0.046
Emotional function	6.10 ± 0.91	6.52 ± 0.68	0.20 (0-0.70)	0.003
Environmental stimuli	5.81 ± 1.08	5.81 ± 1.38	0.00 (-0.75-0.75)	0.931
B-EOS%, %	3.8 (1.5-5.6)	2.6 (1.2-3.5)	-0.03 (-0.12-0.01)	0.002
BEC, 10^9^/L	0.23 (0.08-0.31)	0.16 (0.08-0.20)	-0.50 (-1.85-0.05)	0.007
tIgE, KU/L	150 (17-125)	137 (17-106)	-4.1 (-15.0-1.2)	0.049
sIgE, KU/L	3.44 (0.04-2.74)	3.45 (0.05-2.48)	0.01 (-0.21-0.02)	0.904
FeNO, ppb	44.5 (18.0-48.0)	23.9 (16.0-28.0)	-9.0 (-34.5-2.0)	0.003
FVC, L	3.91 ± 0.88	3.89 ± 0.84	0 (-140-85)	0.486
FVC%pred, %	112.7 ± 10.0	113.1 ± 10.5	0 (-2.80-3.15)	0.755
FEV_1_, L	3.01 ± 0.76	3.03 ± 0.72	0 (-95-150)	0.493
FEV_1_%pred, %	102.5 ± 11.1	104.2 ± 11.1	0 (-2.70-5.60)	0.244
FEV_1_/FVC, %	76.8 ± 6.6	77.7 ± 6.0	0.78 (-0.57-2.42)	0.070

Values are mean±SEM or Median (25th–75th percentile) or *n* (%) unless otherwise stated. ACT: Asthma Control Test; ACQ: Asthma Control Questionnaire; AQLQ: Asthma Quality of Life Questionnaire; B-EOS%: blood eosinophil percent; BEC: blood eosinophil count; tIgE: total immunoglobulin E; sIgE: specific immunoglobulin E; FeNO: fractional exhaled nitric oxide; FVC: forced vital capacity; FVC%pred: forced vital capacity in percent of the predicted value; FEV_1_: forced expiratory volume in 1 second; FEV_1_%pred: forced expiratory volume in one second in percent of the predicted value; FEV_1_/FVC: ratio of forced expiratory volume in 1 second to forced vital capacity.

**Figure 3:**
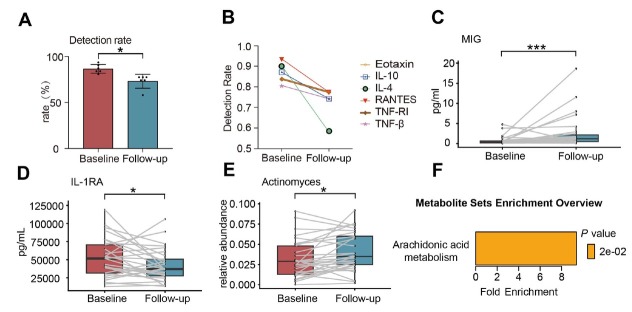
Differences in sputum cytokines, airway microbiota, and plasma metabolites in asthma patients before and after treatment. (A-B) Changes in the detection rates of six cytokines (Eotaxin, IL-10, IL-4, RANTES, TNF-RI, TNF-β) before and after treatment. (C-D) Differential cytokines before and after treatment. (E) Microbial abundance differences before and after treatment. (F) Metabolite enrichment analysis. ^*^P < 0.05; ^***^P < 0.001; IL: interleukin; TNF: tumor necrosis factor; MIG: monokine induced by interferon-gamma.

After ICS treatment, the detection rates of Eotaxin, IL-10, IL-4, RANTES, TNF-RI, and TNF-β in sputum supernatants were below the 80% threshold in all patients and were consequently excluded from subsequent analyses. Post-treatment analysis demonstrated significantly reduced detection rates for all six cytokines compared to their baseline levels (Figure 3A). IL-4 exhibited the most pronounced decrease, followed by RANTES and IL-10 (Figure 3B). Of the 25 cytokines analyzed, only IL-1RA and MIG expressions demonstrated statistically significant changes following ICS treatment (Supplementary Table S2, Figure 3C-3D).

Neither alpha-diversity nor beta-diversity of airway microbiota showed significant changes following ICS treatment (Supplementary Figure S6A-S6H). Analysis of the top ten bacterial genera revealed treatment-associated compositional shifts: *Fusobacterium*, *Neisseria*, and *Prevotella* abundances decreased, while *Actinomyces*, *Rothia*, and *Streptococcus* were increased (Supplementary Figure S6I). Only *Actinomyces* demonstrated statistically significant changes in abundance following treatment (Figure 3E). The metabolic profiles experienced only minimal treatment-induced changes, though several differential metabolites were identified (Supplementary Figure S7A-S7F). Pathway analysis demonstrated significant enrichment of these metabolites in arachidonic acid metabolism (Figure 3F, Supplementary Figure S7G).

### Characterization of responses to ICS treatment in stratified asthma subgroups

Post-treatment analysis revealed significant improvement in ACT scores in all three groups. FeNO levels decreased significantly in ASH and ASM groups. In ASH group AQLQ symptom domain scores were improved, while in ASM group AQLQ emotional function scores were increased. In the ASH group, both blood eosinophil count and the percentage decreased, but lung function improved, as indicated by elevated FEV_1_%pred and FEV_1_/FVC (Supplementary Table S3).

**Figure 4:**
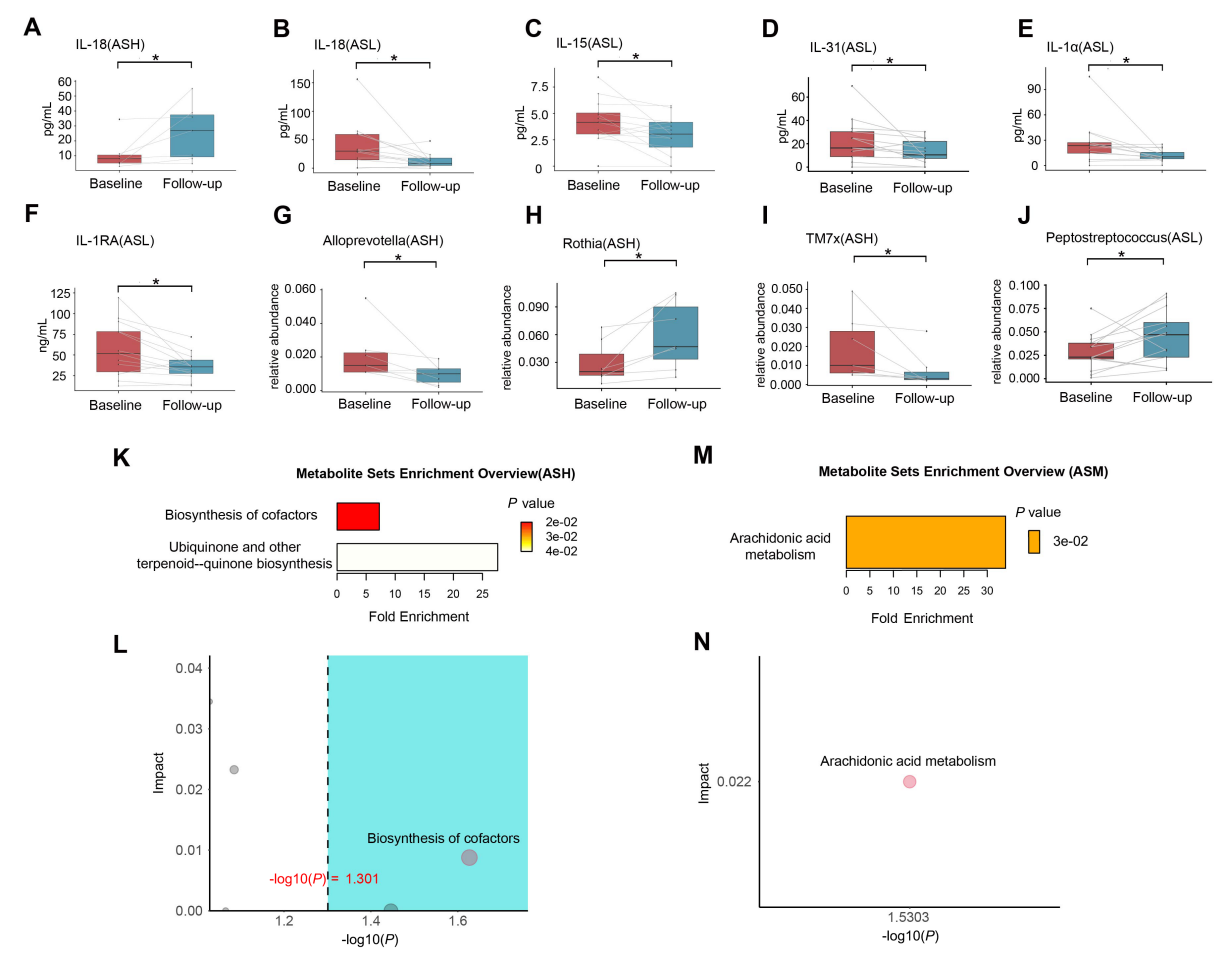
Differences in sputum cytokines, airway microbiota, and plasma metabolites in asthma patients before and after treatment in each subgroup. (A) Differential cytokines in the ASH subgroup. (B-F) Differential cytokines in the ASL subgroup. (G-I) Differential microbial abundances in the ASH subgroup. (J) Differential microbial abundances in the ASL subgroup. (K-L) Metabolite pathway enrichment analysis and topological analysis for the ASH subgroup. (M-N) Metabolite pathway enrichment analysis and topological analysis for the ASM subgroup. ^*^P < 0.05. ASL: FeNO level low; ASM: FeNO level moderate; ASH: FeNO level high; IL: interleukin.

Comparison of sputum supernatant cytokine levels before and after treatment revealed an increase in IL-18 in the ASH group. In the ASL group, IL-6, IL-7, IL-8, IL-15, and IL- 18 levels were decreased, whereas no significant changes in cytokines were observed in the ASM group (Figure 4A-4F).

No significant differences in airway microbiota composition or diversity were found among groups before or after treatment. Differential bacterial analysis showed distinct taxonomic changes across subgroups. In ASH group, *Prevotella* and *TM7x* abundances decreased while *Rothia* increased. In ASL group, *Streptococcus* abundance was increased, whereas no significant microbial changes were observed in ASM group (Figure 4G-4J).

Metabolic profiles remained stable across all groups following ICS treatment, with no significant treatment-associated alterations observed. KEGG enrichment analysis showed that differential metabolites in the ASH group were significantly enriched in the biosynthesis of cofactors and the biosynthesis pathways of ubiquinone and other terpenoid-quinones. Topological analysis revealed that the cofactor biosynthesis pathway was most significantly impacted by differential metabolites (Figure 4K-4L). Metabolites in ASM group were significantly enriched in arachidonic acid metabolism, which was topologically identified as the most impacted pathway (Figure 4M-4N). No metabolic pathways showed significant enrichment in ASL group.

## Discussion

This prospective cohort study multidimensionally investigated the pathological characteristics, intrinsic heterogeneity of, and effects of ICS treatment on T2-high asthma patients. We described the clinical and microscopic features, such as airway cytokines, microbiota composition, and plasma metabolism, in treatment-naive T2-high asthma patients at diagnosis. By hierarchically clustering T2 inflammation biomarkers, we stratified patients in terms of airway inflammation burden, measured by baseline FeNO levels, and systematically compared intergroup differences. Finally, we multidimensionally evaluated the effects of ICS treatment on pathological characteristics across T2-high asthma groups. Our results demonstrated that T2-high asthma patients had distinct airway microbiota signatures, immune response profiles, and plasma metabolic patterns. While these features showed remarkable consistency within the T2-high group, ICS treatment elicited differential responses across patient groups, suggesting that therapeutic effects were subgroup-specific.

While T2 inflammation mechanistically underlies asthma in approximately 50% of mild-to-moderate asthma cases and the most of severe cases, in current clinical practice, standardized criteria are lacking for reliably identifying T2-high asthma subtypes. Although the GINA guidelines for identifying T2-high asthma characteristics were originally developed for severe asthma populations, emerging evidence suggests they are potentially applicable across a broader spectrum of disease severity. Based on this notion, we applied the standardized GINA criteria to our study population.^[1]^ BEC and FeNO are two most utilized clinical biomarkers for T2-high asthma, reflecting distinct but complementary pathways in type 2 inflammation. These biomarkers demonstrated consistent detection rates over a wide array of disease states, from stable asthma to acute exacerbations, and correlate with varying intensities of T2-high inflammation. Asthma patients stratified in terms of BEC and FeNO levels demonstrated significant heterogeneity in clinical manifestations, including symptom burden, disease control status, exacerbation risk profiles, treatment response patterns, and long-term outcomes. The observed phenotypic heterogeneity likely arose from different burdens of T2 inflammation across patient groups.^[18, 19, 20]^ Therefore, we combined BEC and FeNO measurements to conduct hierarchical clustering of T2-high asthma patients, thus allowing for identification of distinct subgroups with varying T2 inflammation burdens. Our results demonstrated that FeNO levels effectively stratified patients into three distinct groups. While FeNO has not been found to be universally correlated with T2 inflammation intensity, growing evidence suggests its elevation may reflect enhanced T2 immune activation. This supports the likelihood of variation of T2 inflammation burdens among the identified groups.^[21,22]^

Consistent with previous findings, our study confirmed that T2-high asthma patients suffered from significant airflow limitation compared to healthy controls. While the ASH group exhibited numerically lower FEV_1_ and FEV_1_/FVC values compared to the other two groups, no statistically significant differences in baseline lung function profiles were observed among the three groups. Previous studies have reported differences in FEV_1_/FVC among asthma patients with varying FeNO levels, and FeNO levels have been found to be intimately associated with symptom control and lung function.^[23,24]^ Notably, the ASH group scored highest on the ACQ scale, indicating potentially worse symptom control and lung function impairment compared to the other groups. Following ICS treatment, we observed significant reductions in FeNO, BEC, and IgE levels, accompanied by complete absence of disease exacerbation or symptom progression. These findings demonstrated that short-term ICS therapy efficatiously attenuated airway inflammation in this population. The lack of significant lung function improvement after ICS treatment may be attributable to well-preserved baseline pulmonary function of the study population and the relatively short treatment duration. The ASH group demonstrated the most pronounced therapeutic response to ICS, as evidenced by the greatest reduction in FeNO and BEC, accompanied by superior symptom control and lung function improvement compared to the other groups. These results are coincident with the PRISM study findings, demonstrating that the magnitude of FeNO and eosinophil reduction was directly correlated with lung function improvement. This reinforces the notion that ICS therapeutic efficacy varies according to baseline airway T2 inflammation burden in asthma patients.^[25]^

Our analysis revealed significant alterations in inflammatory cytokine profiles within the induced sputum of asthma patients, underscoring that this condition has a distinct feature of airway inflammatory microenvironment. At baseline, the three groups showed comparable inflammatory cytokine levels, suggesting they all had similar inflammatory microenvironments. However, our inability to detect key cytokines, including T2-associated IL-4, IL-5, and IL-13, as well as non-T2 cytokines IL-17 and IFN-γ, due to their concentrations being below detection limits, was a significant study limitation. Following ICS treatment, we observed significantly reduced detection rates for six cytokines. This finding is in line with previous studies demonstrating that decreased cytokine concentrations were associated with clinical improvement after ICS therapy, suggesting a treatment-related suppression of inflammatory pathways. Following ICS treatment, we observed only two significant alterations: upregulated MIG (CXCL9) expression and downregulated IL-1RA expression. CXCL9, a Th1-associated chemokine, is primarily secreted by airway epithelial cells following IFN-γ stimulation.^[26]^ Multiple studies have demonstrated elevated sputum CXCL9 levels during asthma exacerbations, with particularly pronounced increase observed in eosinophilic asthma patients compared to their non-eosinophilic counterparts.^[27,28]^ Our results contrast with a recent report by Hastie *et al*., which documented significantly lower CXCL9 concentrations in bronchoalveolar lavage fluid (BALF) in severe asthma patients as compared to their mild-to-moderate counterparts.^[28]^ These conflicting findings highlight the need for further investigation into CXCL9's precise role in asthma pathogenesis and its potential as a therapeutic target. Severe asthma patients exhibited elevated IL-1RA levels in both sputum and BALF compared to non-severe cases. The subsequent decrease in IL-1RA following treatment may indicate alleviated airway inflammation.^[28]^ While ICS treatment differentially modulated cytokine levels across groups in this study, the short duration and extensive data missing limited a definitive interpretation. Future research should cover extended ICS treatment periods and used matched longitudinal biospecimens to obtain more robust findings.

Previous studies have consistently demonstrated elevated airway microbiota diversity and microbial load in asthma patients compared to healthy controls.^[29]^ The identification of multiple differentially abundant bacterial taxa suggests significant alterations in both the composition and organization of airway microbiota in asthma. Consistent with prior reports, our study identified *Actinomyces*, *Fusobacterium*, *Streptococcus*, *Rothia*, and *Neisseria* as the predominant genera in induced sputum from treatment-naive asthma patients, confirming the characteristic airway microbiota profile in this population.^[30,31]^ Of note, our analysis revealed no significant differences in airway microbiota composition or diversity metrics across the three subgroups. While previous studies have extensively characterized microbiota variations across asthma subtypes, the microbial heterogeneity within T2-high asthma populations remains unexplored.^[32]^ Our analysis revealed significantly higher ACE diversity indices and *Granulicatella* abundance in the ASH group relative to ASL group. Current evidence indicates that severe T2-high asthma patients exhibit both elevated airway microbiota α-diversity and increased *Granulicatella* abundance compared to their T2-low counterparts.^[32,33]^ These observations suggest the ASH patients possess a distinct microbial profile that may contribute to its unique pathophysiology. Following ICS treatment, we observed no significant changes in airway microbiota diversity among T2-high asthma patients, which is consistent with prior reports demonstrating microbiome stability after corticosteroid therapy.^[30]^ After treatment, *Actinomyces* abundance was increased significantly. Asthma patients typically have lower *Actinomyces* abundance compared to healthy controls, and bronchial *Actinomyces* abundance has been shown to be negatively correlated with the expression of inflammatory genes.^[34,35]^ Given its potential airway-protecting function, the observed *Actinomyces* expansion following ICS treatment may represent a restoration of beneficial microbiota. Furthermore, we observed subgroup-specific microbial responses to ICS treatment, suggesting that both baseline airway microbiota composition and ICS therapeutic effects may vary with T2 inflammation burden.

Plasma metabolomic profiling has identified significant alterations in asthma patients, with arginine biosynthesis and other amino acid metabolic pathways emerging as prominently enriched ones among differentially expressed metabolites. Dysregulated amino acid metabolism contributes significantly to asthma pathogenesis. Specifically, arginine metabolism modulates Th2 cell activity and functions as a key regulator of T2 airway inflammation.^[36]^ Furthermore, recent metabolomic studies have identified additional perturbations in proline, tyrosine, histidine, lysine, and methionine metabolic pathways in asthma.^[37]^ A metabolomic comparison demonstrated significant enrichment of subgroup-specific differential metabolites in ubiquinone and terpenoid-quinone biosynthesis pathways. Although oxidative stress is known to critically contribute to airway inflammation, particularly during acute exacerbations,^[38]^ we found minimal functional impact of these metabolic variations on oxidative stress pathways. Consequently, these metabolic differences between groups were not considered biologically significant. Our analysis revealed no substantial differences in baseline metabolic profiles among T2-high asthma subgroups. We subsequently characterized the metabolic changes induced by ICS treatment within this population. ICS-induced alterations in lipid metabolism were found to be a key area of investigation. Our study demonstrated significant reductions in multiple plasma lipid species following ICS treatment in T2-high asthma patients, including phosphatidylcholine (PC), phosphatidylinositol (PI), eicosatetraenoic acid (ETE), hydroxyeicosatetraenoic acid (HETE), and prostaglandins. PC and PI are essential glycerophospholipid constituents of cellular membranes.^[39]^ Metabolomic profiling of sputum has revealed significant alterations involved in glycerophospholipid pathway among asthma patients.^[40]^ Gai *et al*. demonstrated serum glycerophospholipid levels were elevated in eosinophilic asthma patients relative to their non-eosinophilic counterparts.^[41]^ Arachidonic acid (AA), an omega-6 polyunsaturated fatty acid, is an essential structural component of cell membrane phospholipid bilayers.^[42]^ AA is metabolized through oxidative pathways to generate lipoxygenase, cytochrome P450, and cyclooxygenase products, which are subsequently converted into potent inflammatory mediators, including leukotrienes and prostaglandins. These metabolites activate mast cells and group 2 innate lymphoid cells (ILC2s) while promoting eosinophil recruitment.^[43,44]^ Notably, serum AA concentrations are elevated in severe asthma patients but are significantly reduced following ICS treatment.^[45,46]^ After treatment, multiple AA metabolites, including ETE, HETE, and prostaglandins were significantly lowered. Notably, treatment-associated differential metabolites were significantly enriched in arachidonic acid metabolism pathways (FDR < 0.05), demonstrating ICS-mediated rectification of lipid metabolic dysregulation in asthma. However, we observed subgroup-specific metabolic response patterns, indicating that the ICS effects vary across patient populations.

In conclusion, our study demonstrated that the clinical manifestations of T2-high asthma vary significantly, which fully indicates a strong heterogeneity of this disease. Although the differences among T2-high subgroups at baseline were relatively minor, the effects of ICS treatment on each subgroup were conspicuously different. This subgroup-specific response potentially holds important implication in clinical practice. For instance, by taking into consideration the differences among different patient groups, the optimal dosage and treatment course of ICS can be determined to improve the therapeutic efficacy and reduce unnecessary adverse drug reactions. Further clarifying the characteristic responses of different patient groups to ICS is conducive to the accurate prediction of the disease progression and prognosis. In addition, future research can delve into the underlying mechanisms of the differential responses of different patient groups to ICS, and help develop more specific and efficacious treatment to maximally benefit asthma patients.

This study is subject to several limitations. Firstly, the relatively small cohort of T2-high asthma patients, particularly in individuals subgrouped in terms of FeNO level, statistically limited our power to detect significant differences in baseline characteristics and longitudinal changes. Future multi-center studies involving large sample sizes are warranted to improve the representativeness of the samples and the generalizability of the research findings. Secondly, our airway proteomic analysis failed to detect some relevant cytokines, and the lack of corresponding plasma proteomic data represents a significant methodological limitation. Thirdly, the relatively short follow-up duration in this study precluded evaluation of long-term ICS treatment effects in T2-high asthma patients stratified by airway inflammation severity. It is necessary to conduct prospective cohort studies and longterm follow-ups of patients, and to holistically assess the short- and long-term effects, safety, and the impact of ICS treatments on patients‘ quality of life. Fourthly, our airway microbiome analysis exclusively focused on bacterial taxa and failed to cover fungal communities. Larger-scale studies incorporating both bacterial and fungal profiling are needed to validate these observations. Finally, due to the observational nature of this study and the limitations inherent to the study design and sample size, we are currently unable to conduct a more in-depth examination into the underlying mechanisms of the subgroup-specific responses to ICS treatment. However, we believe that the findings of this study laid a foundation for future research.

## Conclusion

By integrating microbiomic, proteomic, and metabolomic analyses, this study characterized T2-high asthma heterogeneity across the spectrum of airway inflammation severity and confirmed the effects of longitudinal ICS treatment. Our study multidimensionally identified distinct pathological characteristics in treatment-naive T2-high asthma patients at diagnosis, though intergroup heterogeneity in terms of airway inflammation burden was limited. ICS treatment effectively improves abnormal airway microenvironments and rectifies lipid metabolism dysregulation in T2-high asthma patients, although its therapeutic effects vary significantly across different patient groups with different levels of T2 inflammation burden. This study significantly advances asthma omic research by elucidating the heterogeneity of T2-high asthma and delineating subgroup-specific effects of ICS treatment, providing valuable insights for understanding disease heterogeneity.

## Supplementary Material

Supplementary Material Details
