# Supplementary material for: Effects of T2-high asthma heterogeneity and inhaled corticosteroid on airway and metabolic profiles: A multi-omic approach
Source: J Transl Int Med. 2026 Feb 13;14(1):79–95. doi: 10.1515/jtim-2026-0001 (PMC12916266; doi:10.1515/jtim-2026-0001)
Supplement: Supplementary file 1 — Supplementary Material Details [file jtim-2026-0001_sm.pdf]

Supplementary materials

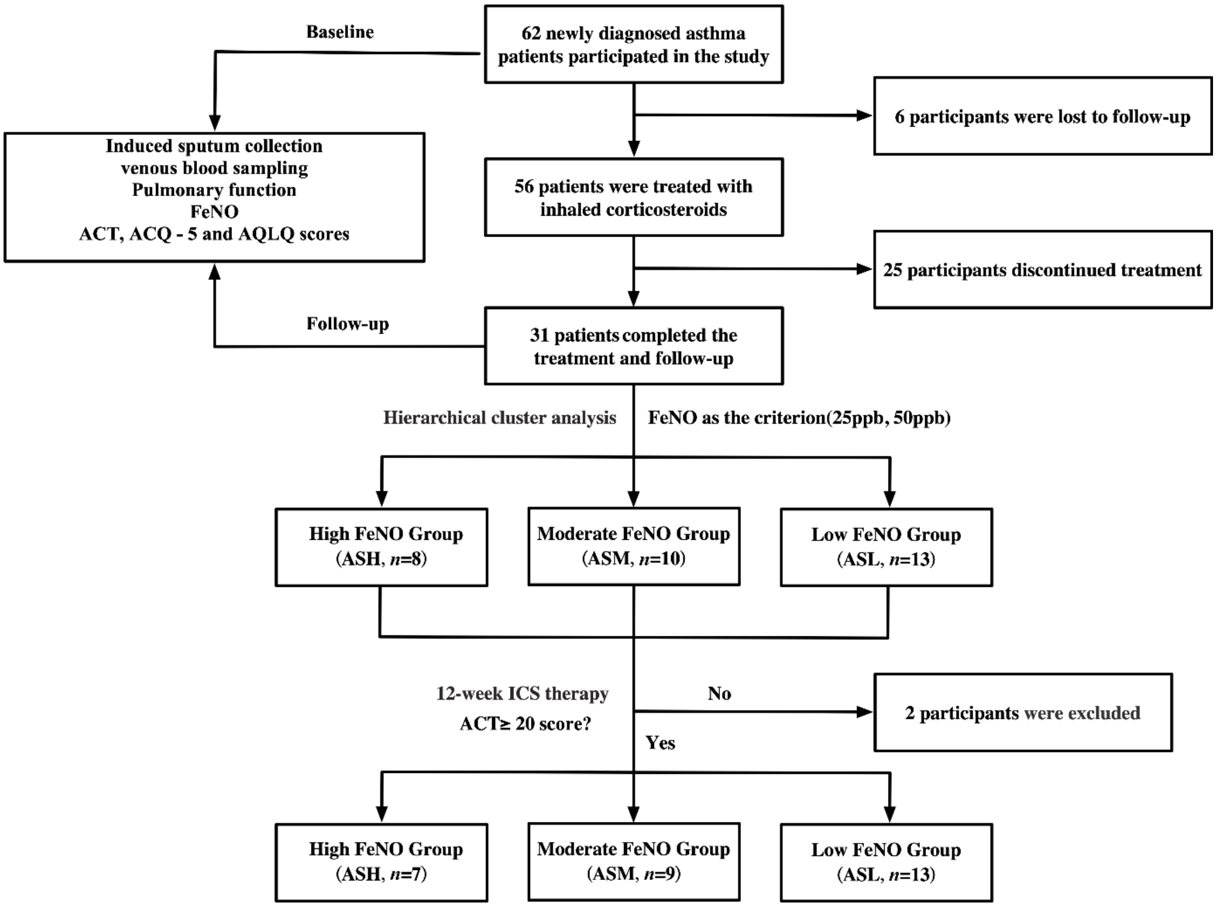

Supplementary Figure S1: Flowchart of the asthma patient study. ACT: Asthma Control Test; ACQ: Asthma Control Questionnaire; AQLQ: Asthma Quality of Life Questionnaire; ASH: asthma FeNO level high; ASL: asthma FeNO level low; ASM: asthma FeNO level moderate; FeNO: fractional exhaled nitric oxide; ICS: inhaled corticosteroids.

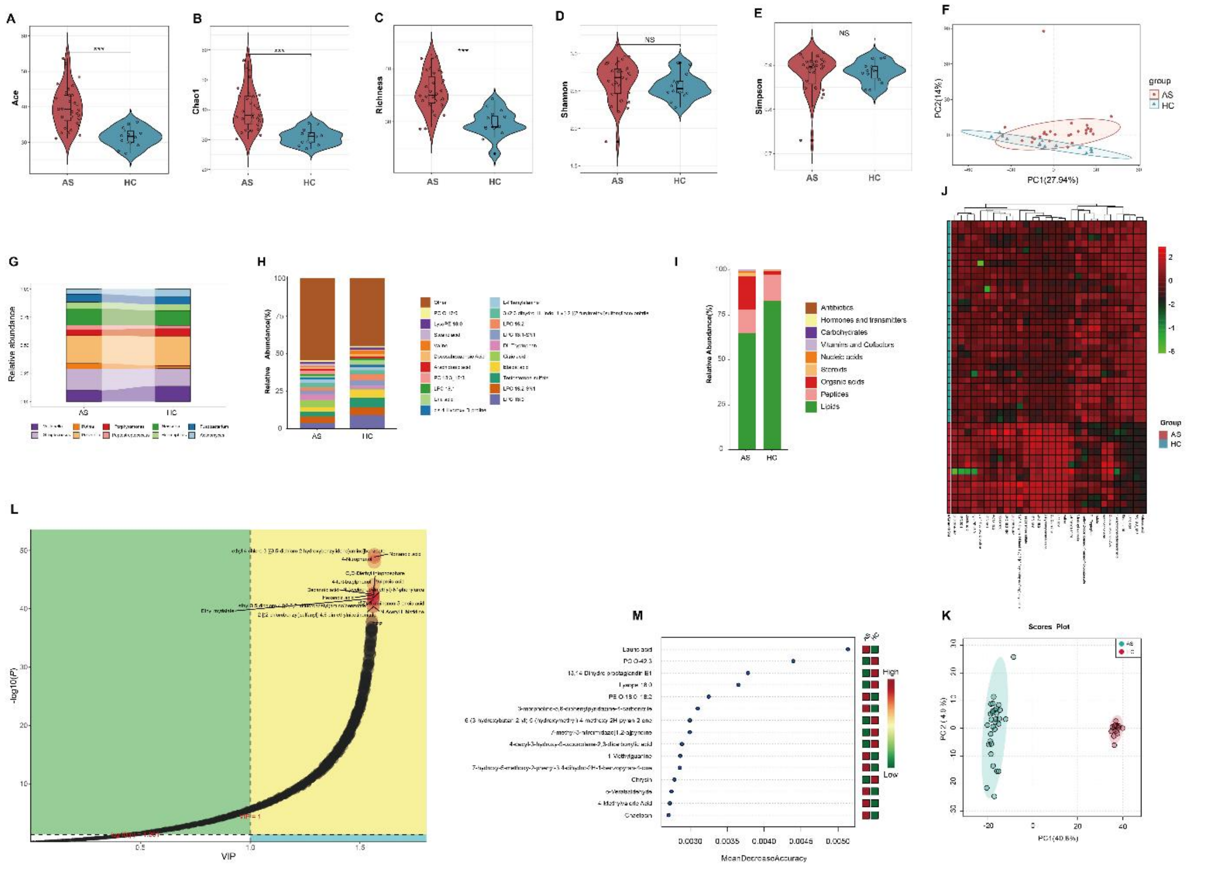

Supplementary Figure S2: Comparison of airway microbiota and plasma metabolites between T2-high asthma patients and healthy controls at baseline. (A-E) Comparison of airway microbiota α-diversity between asthma patients and healthy controls. (F) Comparison of airway microbiota β-diversity between asthma patients and healthy controls. (G) Sankey diagram showing the differences in the abundance of the top ten bacterial species between the two groups. (H) Stacked bar chart showing the proportion of the top 20 metabolites in the AS group and HC group. The order from top to bottom corresponds to the metabolites listed in the legend. (I) Stacked bar chart showing the percentage of different types of metabolites in the AS group and HC group. (J) Heatmap clustering analysis of metabolites. The y-axis includes sample names and corresponding group information, and the x-axis represents metabolites. The clustering tree at the top shows the similarity in metabolite distribution between samples, while the heatmap in the middle denotes metabolite content, with color shades reflecting the Z-score values. (K) PCA analysis comparing the AS group and HC group. Each point represents a sample, and the distance between points indicates differences in metabolite composition (Euclidean distance). Different groups are represented by different colors, with ellipses indicating the 95% confidence interval for sample points. (L) PLSDA analysis of metabolite importance. Each point represents a metabolite, with the x-axis showing the VIP score, and the y-axis displaying the FDR-corrected *P*-value (log10 transformed). (M) Random forest analysis showing the top 15 most important metabolites. The x-axis represents the "mean decrease accuracy" metric, reflecting the importance of each metabolite in the random forest model. The right panel displays a heatmap of the abundance of these 15 metabolites between the two groups. Ns, $P > 0.05$; $^{***}P < 0.001$. AS: asthma patients; HC: healthy control; PC: principal coordinates; PLSDA: partial least squares discriminant analysis; VIP: variable importance in projection.

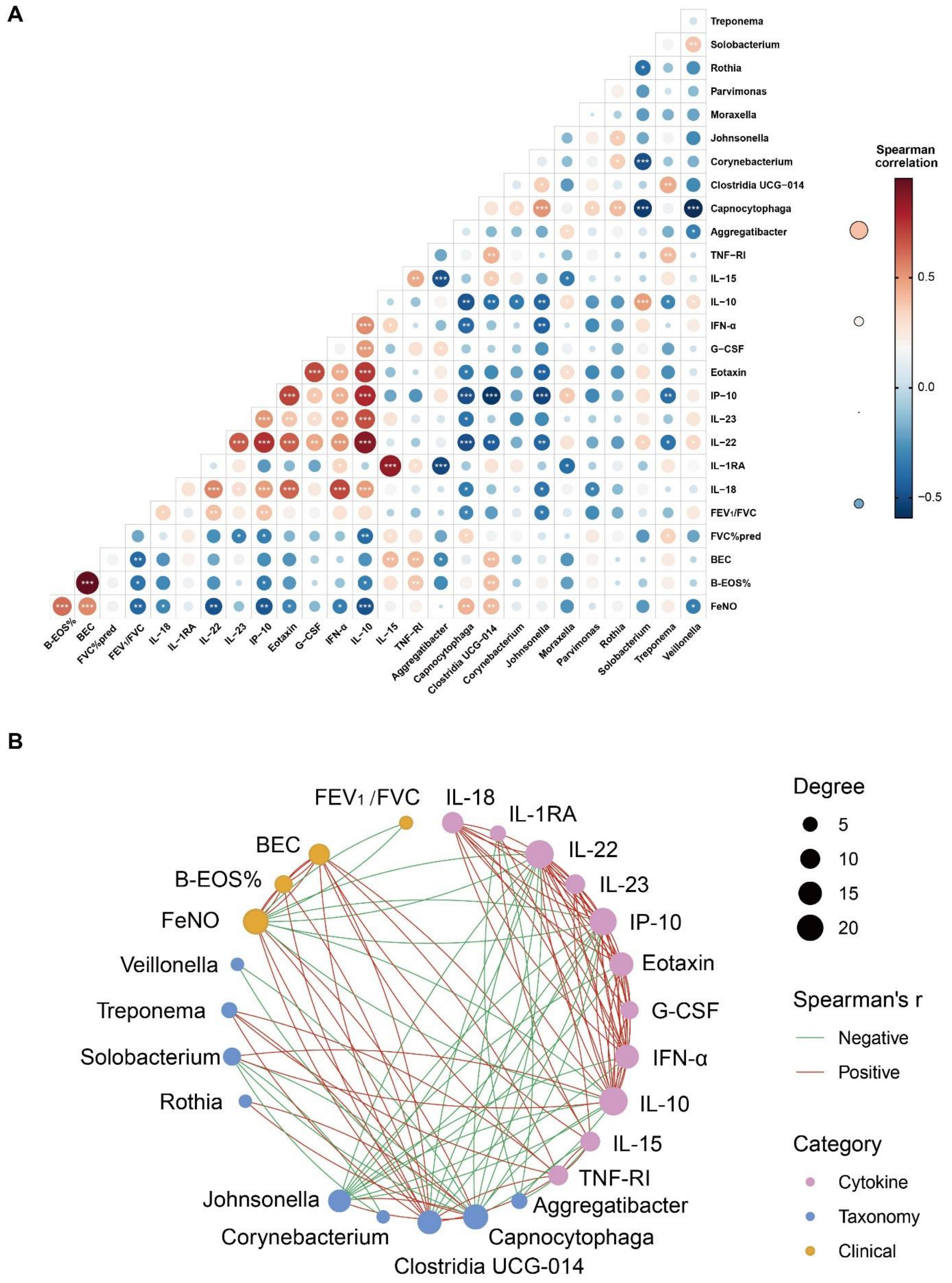

Supplementary Figure S3: Correlation plot of differential indicators between T2-high asthma patients and healthy controls at baseline. (A) Spearman correlation heatmap showing the correlations between differential clinical indicators, cytokines, and microbiota community indicators in healthy controls and T2-high asthma patients. The size of each dot represents the strength of the Spearman correlation between two variables. Statistical significance is marked

within the dots. The color indicates the direction and strength of the correlation, as shown by the color scale on the right-red represents a positive correlation, blue represents a negative correlation, with darker colors indicating stronger correlations (absolute value closer to 1) and lighter colors indicating weaker correlations (closer to 0). (B) Network diagram based on Spearman correlation. Nodes represent different indicators, with purple nodes for cytokines, blue nodes for microbiota, and yellow nodes for clinical indicators. The size of each node is determined by its Degree, with larger nodes indicating more connections to other variables. The lines between nodes represent correlations, with green lines indicating negative correlations and red lines indicating positive correlations. The connections and node sizes visually display the association patterns and the strength of connections between variables. $^{*}P < 0.05$; $^{**}P < 0.01$; $^{***}P < 0.001$.

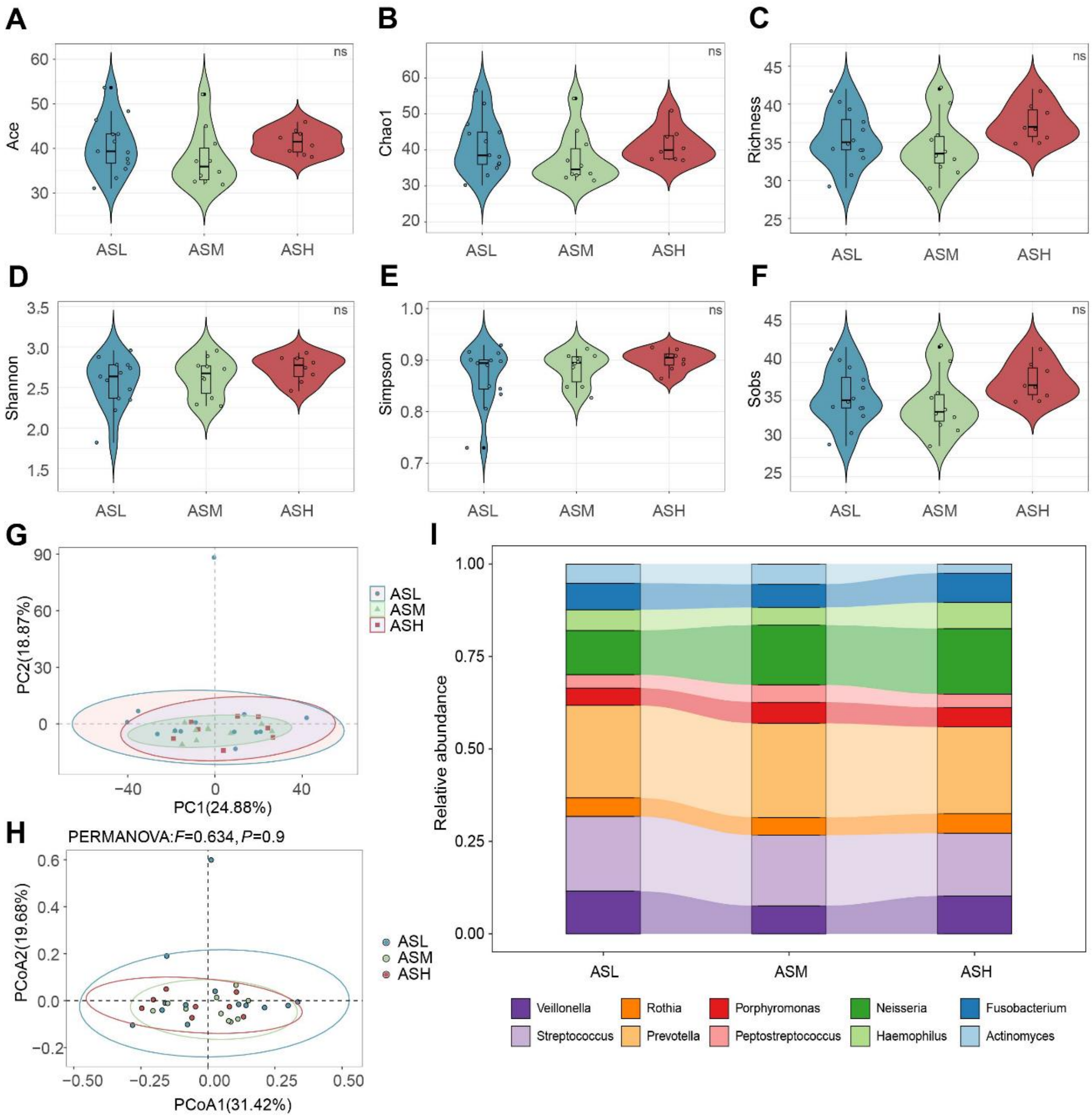

Supplementary Figure S4: Comparison of airway microbiota diversity and structure in T2-high asthma subgroups at baseline. (A-F) Comparison of airway microbiota α-diversity among subgroups. (G-H) Comparison of airway microbiota β-diversity among subgroups. (I) Sankey diagram showing the differences in the abundance of the top ten bacterial species between the

three groups. ns, $P > 0.05$. ASH: asthma FeNO level high; ASL: asthma FeNO level low; ASM: asthma FeNO level moderate.

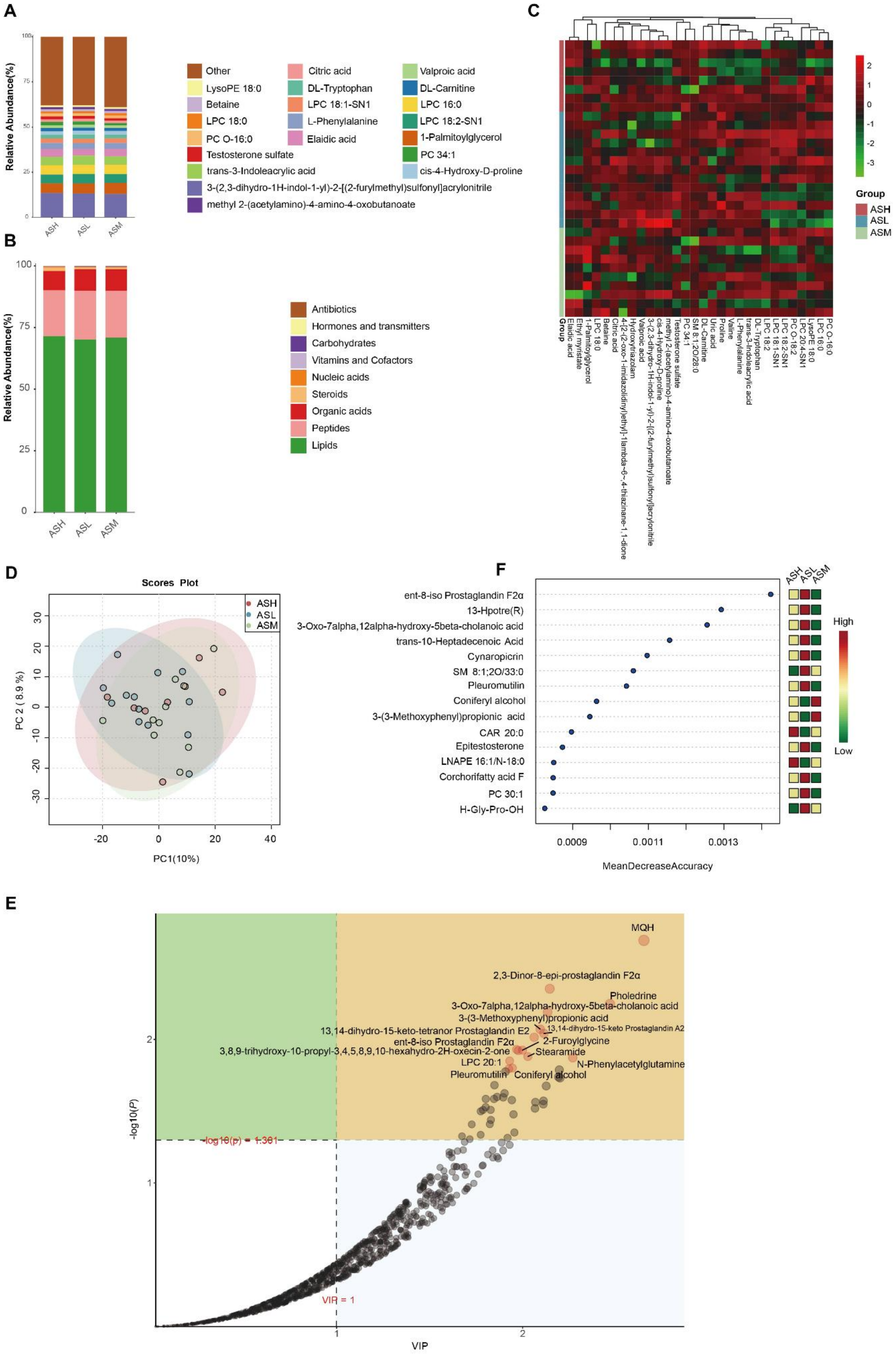

A
B
C
D
E
F
Relative Abundance(%)
ASH
ASL
ASM
Other
LysoPE 18:0
Betaine
LPC 18:0
PC O-16:0
Testosterone sulfate
trans-3-Indoleacrylic acid
3-(2,3-dihydro-1H-indol-1-yl)-2-[(2-furylmethyl)sulfonyl]acrylonitrile
methyl 2-(acetylamino)-4-amino-4-oxobutanoate
Citric acid
DL-Tryptophan
LPC 18:1-SN1
L-Phenylalanine
Elaidic acid
Valproic acid
DL-Carnitine
LPC 16:0
LPC 18:2-SN1
1-Palmitoylglycerol
PC 34:1
cis-4-Hydroxy-D-proline
Antibiotics
Hormones and transmitters
Carbohydrates
Vitamins and Cofactors
Nucleic acids
Steroids
Organic acids
Peptides
Lipids
Group
Scores Plot
PC1(10%)
PC 2 (8.9 %)
ent-8-iso Prostaglandin F2α
13-Hpotre(R)
3-Oxo-7alpha,12alpha-hydroxy-5beta-cholanoic acid
trans-10-Heptadecenoic Acid
Cynaropicrin
SM 8:1;2O/33:0
Pleuromutilin
Coniferyl alcohol
3-(3-Methoxyphenyl)propionic acid
CAR 20:0
Epitestosterone
LNAPE 16:1/N-18:0
Corchorifatty acid F
PC 30:1
H-Gly-Pro-OH
MeanDecreaseAccuracy
High
Low
MQH
2,3-Dinor-8-epi-prostaglandin F2α
Pholedrine
13,14-dihydro-15-keto-tetranor Prostaglandin E2
13,14-dihydro-15-keto Prostaglandin A2
2-Furoylglycine
3,8,9-trihydroxy-10-propyl-3,4,5,8,9,10-hexahydro-2H-oxecin-2-one
Stearamide
N-Phenylacetylglutamine
LPC 20:1
VIP = 1
-log10(p) = 1.301
-log10(P)
VIP

Supplementary Figure S5: Comparison of metabolites in T2-high asthma subgroups at baseline. (A) Stacked bar chart showing the percentage of the top 20 metabolites in the three groups. (B) Stacked bar chart showing the percentage of different types of metabolites in the three groups. (C) Heatmap clustering analysis of metabolites in the three groups. (D) PCA analysis of metabolites in the three groups. (E) PLSDA analysis of metabolite importance in the three groups. (F) Random forest analysis showing the top 15 most important metabolites in the three groups. ASH: asthma FeNO level high; ASL: asthma FeNO level low; ASM: asthma FeNO level moderate; PLSDA: partial least squares discriminant analysis.

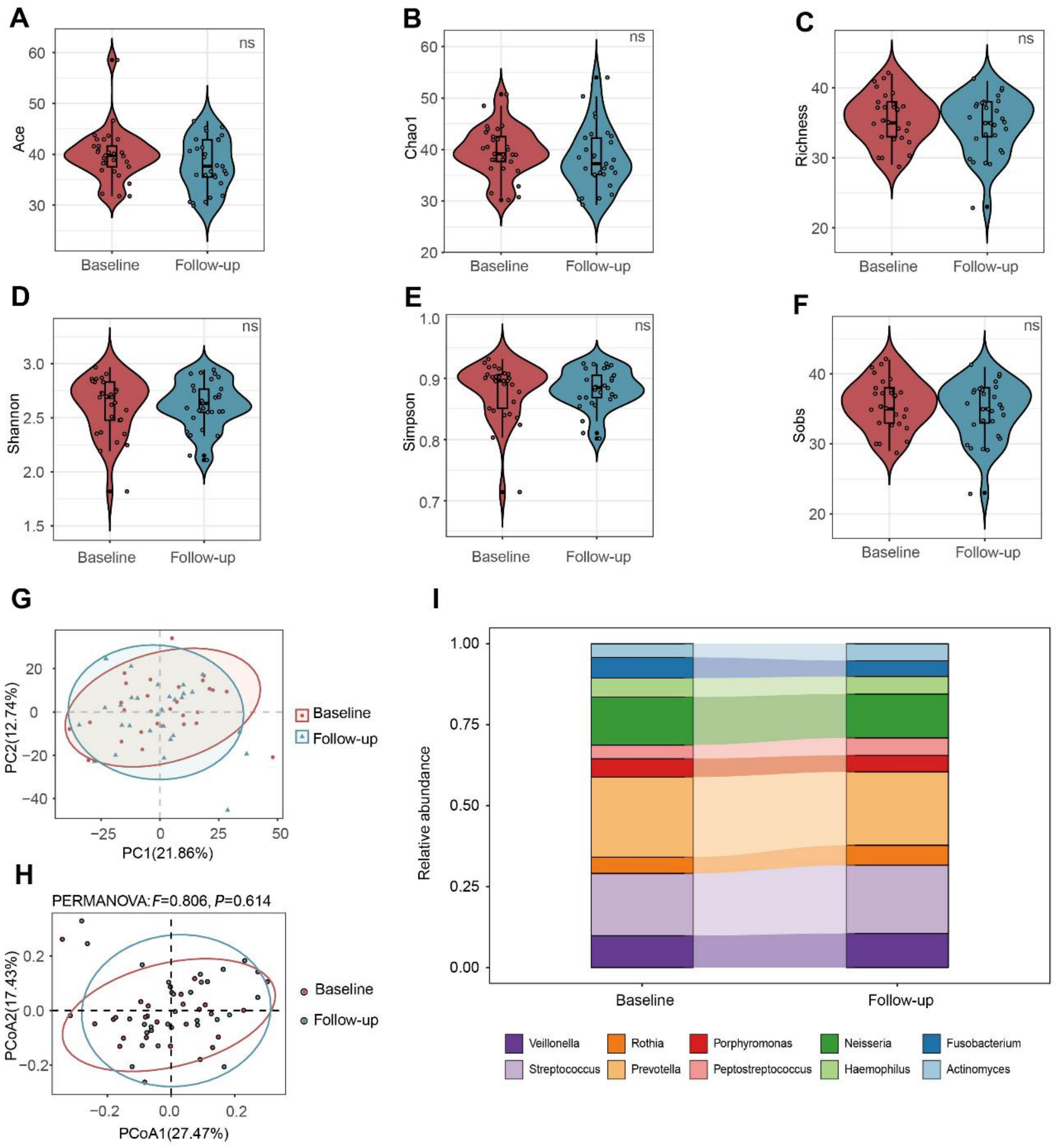

Supplementary Figure S6: Changes in airway microbiota diversity and structure in asthma patients after ICS treatment. (A-F) Comparison of airway microbiota α-diversity. (G-H) Comparison of airway microbiota β-diversity. (I) Sankey diagram showing the differences in the abundance of the top ten bacterial species. ns, $P > 0.05$.

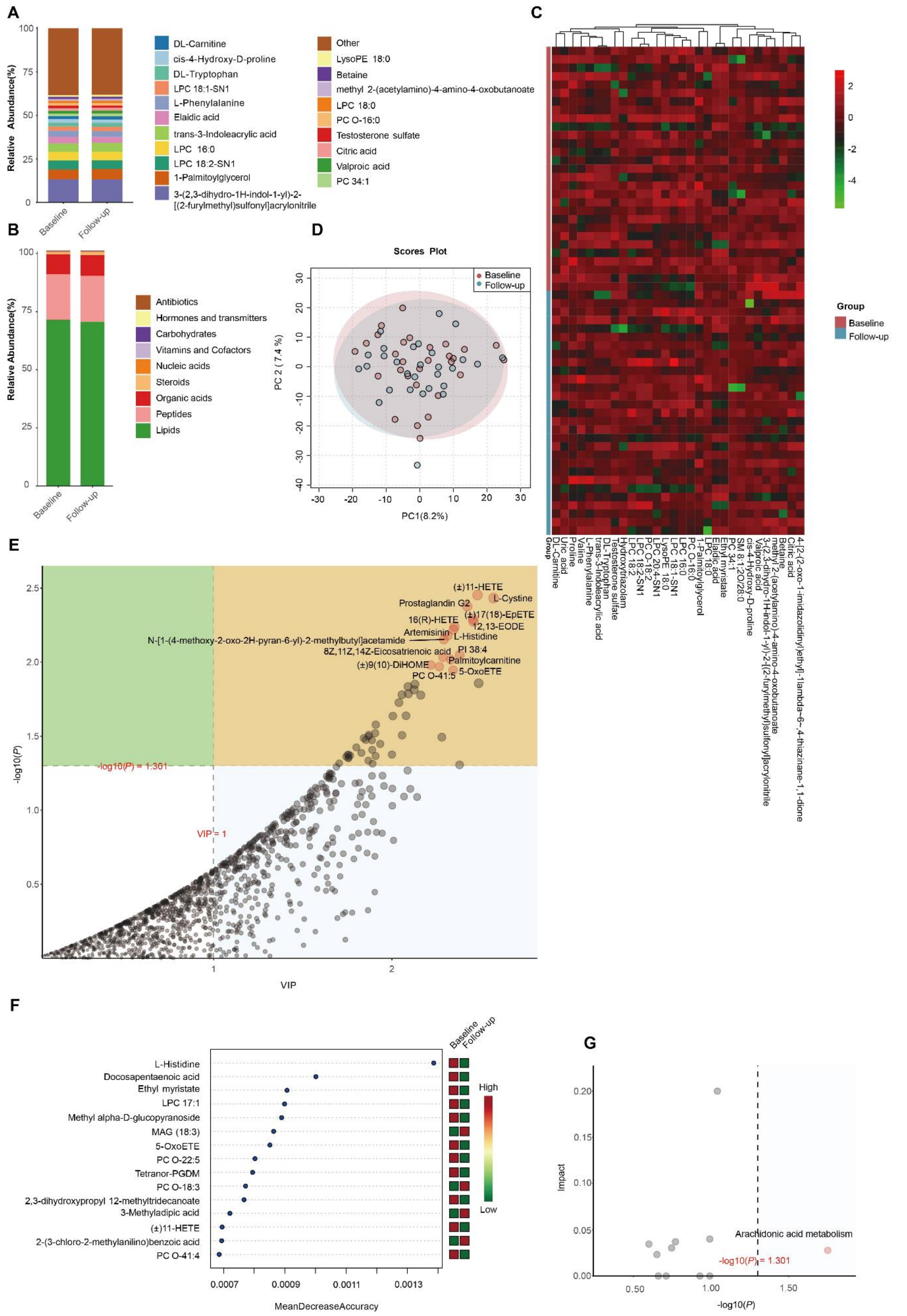

Supplementary Figure S7: Comparison of metabolic differences in asthma patients before and after ICS treatment. (A) Stacked bar chart showing the percentage of the top 20 metabolites.

(B) Stacked bar chart showing the percentage of different types of metabolites. (C) Heatmap clustering analysis of metabolites. (D) PCA analysis of metabolites. (E) PLSDA analysis of metabolite importance. (F) Random forest analysis showing the top 15 most important metabolites. (G) Metabolite enrichment and topological analysis. ICS: inhaled corticosteroids; ASH: asthma FeNO level high; ASL: asthma FeNO level low; ASM: asthma FeNO level moderate; PCA: principal coordinates analysis; PLSDA: partial least squares discriminant analysis.

Supplementary Table S1: Baseline sputum cytokines of three stable FeNO subgroups

Cytokines*	ASH (*n* = 8)	ASM (*n* = 10)	ASL (*n* = 13)	P^{all}	P^{HM}	P^{HL}	P^{ML}
CCL17	0.21 (0.16-0.29)	0.24 (0.20-0.30)	0.18 (0.15-0.21)	0.351	0.605	0.557	0.182
Eotaxin	0.58 (0.26-0.85)	0.83 (0.37-1.14)	0.90 (0.69-1.28)	0.507	0.329	0.166	0.789
Eotaxin-2	1.00 (1.00-1.17)	1.14 (0.94-1.25)	1.10 (1.06-1.22)	0.761	0.428	0.537	0.764
FGF-2	8.98 (6.85-10.84)	6.54 (5.18-8.60)	8.44 (6.40-13.04)	0.229	0.248	0.772	0.577
G-CSF	23.78 (8.26-39.00)	6.85 (6.39-16.94)	30.32 (6.39-31.05)	0.647	0.534	1.000	0.383
IFN-α	0.28 (0.17-0.39)	0.38 (0.22-0.54)	0.46 (0.38-0.53)	0.168	0.301	0.050	0.461
IL-10	0.37 (0.02-0.52)	0.39 (0.18-0.54)	0.66 (0.25-0.80)	0.368	0.874	0.244	0.304
IL-15	3.46 (2.32-4.47)	3.86 (3.14-4.07)	4.20 (3.04-5.04)	0.828	0.656	0.445	0.664
IL-18	9.60 (4.29-10.12)	34.05 (23.28-48.83)	38.30 (14.53-59.36)	0.080	**0.029**	0.082	0.975
IL-1RA	42.79 (20.36-58.16)	46.98 (33.38-57.26)	56.6 6(29.63-78.26)	0.634	0.307	0.205	0.733
IL-1α	17.11 (3.15-18.84)	21.75 (9.23-33.27)	26.82 (14.65-27.21)	0.379	0.689	0.538	0.975
IL-1β	155.1 (39.1-223.2)	163.9 (69.3-207.3)	188.7 (56.9-295.0)	0.819	0.708	0.272	0.417
IL-21	6.51 (2.27-10.93)	6.89 (4.19-6.88)	9.34 (5.34-9.96)	0.822	0.853	0.716	0.552
IL-22	9.24 (7.89-9.96)	9.73 (8.88-11.13)	10.00 (9.74-11.03)	0.371	0.518	0.176	0.399
IL-23	4.78 (1.03-8.03)	3.56 (1.37-5.31)	4.55 (1.37-8.03)	0.887	0.500	0.941	0.875
IL-31	17.71 (6.63-23.70)	18.10 (7.42-18.15)	22.4 6(8.96-30.34)	0.685	1.000	0.611	0.401
IL-33	13.22 (12.75-14.69)	13.25 (11.98-14.21)	12.83 (12.54-14.21)	0.730	0.979	0.445	0.999
IL-4	6.50 (6.29-6.57)	5.72 (6.29-6.29)	5.78 (6.29-6.29)	0.680	0.426	0.506	0.971
IL-6	7.58 (6.21-9.37)	10.35 (4.90-12.93)	12.24 (7.84-13.62)	0.545	0.929	0.325	0.437
IL-7	0.50 (0.37-0.61)	0.52 (0.38-0.63)	0.65 (0.47-0.68)	0.658	0.928	0.465	0.472
IL-8	116.8 (43.7-189.8)	151.5 (39.0-152.5)	177.5 (49.4-208.2)	0.676	0.824	0.638	0.402
IP-10	1.09 (1.05-1.32)	1.21 (1.09-1.32)	1.22 (1.19-1.38)	0.654	1.000	0.532	0.410
MIG	0.87 (0.22-0.76)	1.41 (0.34-1.61)	2.63 (0.14-1.28)	0.595	0.422	0.971	0.384
MIP-1α	4.85 (0.76-7.04)	2.38 (0.33-2.71)	1.63 (0.58-1.93)	0.369	0.182	0.405	0.515
MIP-1β	26.64 (7.98-33.65)	22.18 (0.68-32.93)	23.59 (5.65-42.01)	0.740	0.689	0.971	0.437
MMP-9	385.40 (92.50-521.30)	710.40 (229.40-909.60)	517.20 (305.50-679.90)	0.576	0.450	0.397	0.642
RANTES	0.58 (0.55-0.64)	0.58 (0.50-0.60)	0.56 (0.50-0.64)	0.918	0.751	0.739	0.999
TNF-RI	202.30 (170.80-275.90)	148.60 (91.30-190.50)	201.90 (190.50-261.20)	0.333	0.286	0.802	0.437

TNF-RII	0.48 (0.46-0.62)	0.57 (0.52-0.63)	0.54 (0.51-0.66)	0.514	0.299	0.482	0.198
TNF-β	8.34 (6.43-9.69)	5.88 (3.69-9.12)	7.67 (0.00-13.68)	0.626	0.397	0.276	0.950
TSLP	0.78 (0.49-1.08)	0.70 (0.54-0.97)	0.81 (0.70-1.08)	0.784	0.672	0.897	0.450

Values are Median (25th-75th percentile) unless otherwise stated. *The expression levels of other cytokines are presented in pg/mL, while that of IL-RA is in ng/mL. ASH: asthma FeNO level high; ASL: asthma FeNO level low; ASM: asthma FeNO level moderate. CCL: chemokine (C-C motif) ligand; FGF: fibroblast growth factor; G-CSF: granulocyte colony-stimulating factor; IFN: interferon; IL: interleukin; IP: interferon-γ-inducible protein; MIP: macrophage inflammatory protein; MMP: matrix metalloproteinase; TNF-R: tumor necrosis factor receptor.

Supplementary Table S2: Sputum cytokines compared across baseline and follow-up of the enrolled initial-diagnosis asthma patients

Cytokines*	Baseline	Follow-up	Fold change (F/B)	*P*
CCL17	0.20 (0.15-0.27)	0.26 (0.15-0.30)	1.294	0.274
Eotaxin-2	1.08 (0.99-1.24)	1.39 (0.96-1.39)	1.280	0.352
FGF-2	20.98 (6.02-10.03)	9.62 (6.66-11.61)	0.458	0.173
G-CSF	23.03 (6.39-26.85)	33.69 (7.66-54.22)	1.463	0.059
IFN-α	0.38 (0.22-0.48)	0.33 (0.18-0.42)	0.856	0.421
IL-15	4.07 (3.04-5.04)	3.08 (0.86-4.16)	0.756	0.071
IL-18	29.51 (8.08-34.43)	18.90 (6.41-25.38)	0.640	0.198
IL-1α	23.85 (9.10-27.21)	16.10 (6.83-21.82)	0.675	0.066
IL-1β	177.10 (61.70-232.60)	178.80 (59.00-226.50)	1.010	0.574
IL-1RA	52.21 (31.59-70.53)	41.74 (27.67-50.73)	0.799	0.047
IL-21	7.99 (5.34-10.74)	7.18 (3.80-9.96)	0.899	0.590
IL-22	9.87 (8.88-11.03)	9.90 (8.88-11.45)	1.003	0.679
IL-23	4.31 (1.37-6.95)	3.69 (1.37-5.86)	0.858	0.667
IL-31	20.79 (8.96-30.34)	16.38 (7.42-24.84)	0.788	0.548
IL-33	13.03 (11.98-14.21)	12.86 (11.41-14.48)	0.987	0.974
IL-6	9.61 (6.21-10.84)	12.41 (4.46-18.78)	1.292	0.243
IL-7	0.59 (0.42-0.68)	0.55 (0.37-0.68)	0.935	0.903
IL-8	153.30 (45.50-201.40)	154.30 (76.60-175.60)	1.007	0.559
IP-10	1.18 (1.05-1.32)	1.08 (1.05-1.32)	0.916	0.123
MIG	1.71 (0.19-0.87)	5.54 (0.54-2.54)	3.235	0.002
MIP-1α	2.24 (0.44-2.27)	1.89 (0.46-1.67)	0.845	0.545
MIP-1β	21.93 (3.73-33.64)	23.63 (15.39-28.62)	1.077	0.641
MMP-9	536.60 (196.30-752.30)	630.80 (269.60-933.50)	1.175	0.166
TNF-RII	0.53 (0.49-0.63)	0.55 (0.49-0.66)	1.050	0.446
TSLP	0.73 (0.49-1.08)	0.81 (0.49-1.08)	1.108	0.989

Values are Median (25th-75th percentile) unless otherwise stated. *The expression levels of other cytokines are presented in pg/mL, while that of IL-RA is in ng/mL. ASH: asthma FeNO level high; ASL: asthma FeNO level low; ASM: asthma FeNO level moderate. CCL: chemokine (C-C motif) ligand; FGF: fibroblast growth factor; G-CSF: granulocyte colony-stimulating factor; IFN: interferon; IL: interleukin; IP: interferon-γ-inducible protein; MIP: macrophage inflammatory protein; MMP: matrix metalloproteinase; TNF-R: tumor necrosis factor receptor.

Supplementary Table S3: Clinical indicators of variations among T2-high asthma subgroups after ICS treatment

	Baseline	Follow-up	*P*
ASH subgroup (*n* = 7)			
ACT, score	17.25 ± 2.60	22.88 ± 2.10	0.022
ACQ, score	1.90 (0.90-2.80)	0.50 (0.20-0.50)	0.021
AQLQ, score			

Symptoms	4.94 ± 1.07	6.37 ± 0.61	0.022
B-EOS%, %	8.6 (5.9-10.1)	4.4 (3.3-5.3)	0.014
BEC, 10^9/L	0.52 (0.33-0.56)	0.24 (0.19-0.25)	0.014
FeNO, ppb	111.0 (90.3-127.5)	32.8 (15.8-46.3)	0.014
FEV_1%pred, %	92.2 ± 18.7	104.3 ± 8.1	0.021
FEV_1/FVC, %	69.4 ± 10.9	75.0 ± 7.3	0.021
ASM subgroup (*n* = 9)			
ACT, score	19.10 ± 1.37	23.20 ± 1.50	0.006
AQLQ, score	5.73 ± 0.83	6.25 ± 0.81	0.006
Emotional function	5.90 ± 0.96	6.64 ± 0.62	0.009
FeNO, ppb	35.1 (30.3-38.8)	23.1 (18.3-24.8)	0.019
ASL subgroup (*n* = 13)			
ACT, score	18.77 ± 2.74	22.54 ± 1.33	0.002

Values are mean±SEM or Median (25th-75th percentile) unless otherwise stated. ACT: Asthma Control Test; ACQ: Asthma Control Questionnaire; AQLQ: Asthma Quality of Life Questionnaire; B-EOS%: blood eosinophil percent; BEC: blood eosinophil count; FeNO: fractional exhaled nitric oxide; FEV_1%pred: forced expiratory volume in one second in percent of the predicted value; FEV_1/FVC: ratio of forced expiratory volume in 1 second to forced vital capacity. ICS: inhaled corticosteroids; ASH: asthma FeNO level high; ASL: asthma FeNO level low; ASM: asthma FeNO level moderate. CCL: chemokine (C-C motif) ligand; FGF: fibroblast growth factor; G-CSF: granulocyte colony-stimulating factor; IFN: interferon; IL: interleukin; IP: interferon-γ-inducible protein; MIP: macrophage inflammatory protein; MMP: matrix metalloproteinase; TNF-R: tumor necrosis factor receptor.
